# MLKL polymerization-induced lysosomal membrane permeabilization promotes necroptosis

**DOI:** 10.1038/s41418-023-01237-7

**Published:** 2023-11-23

**Authors:** Shuzhen Liu, Preston Perez, Xue Sun, Ken Chen, Rojin Fatirkhorani, Jamila Mammadova, Zhigao Wang

**Affiliations:** 1 Department of Molecular Biology, University of Texas Southwestern Medical Center, 5323 Harry Hines Boulevard, Dallas, TX 75390 USA; 2 Center for Regenerative Medicine, Heart Institute, Department of Internal Medicine, Morsani College of Medicine, University of South Florida, 560 Channelside Drive, MDD714, Tampa, FL 33602 USA; 3 Department of Emergency Medicine, the First Affiliated Hospital of Soochow University, Suzhou, Jiangsu 21500 China

**Keywords:** Lysosomes, Proteolysis

## Abstract

Mixed lineage kinase-like protein (MLKL) forms amyloid-like polymers to promote necroptosis; however, the mechanism through which these polymers trigger cell death is not clear. We have determined that activated MLKL translocates to the lysosomal membrane during necroptosis induction. The subsequent polymerization of MLKL induces lysosome clustering and fusion and eventual lysosomal membrane permeabilization (LMP). This LMP leads to the rapid release of lysosomal contents into the cytosol, resulting in a massive surge in cathepsin levels, with Cathepsin B (CTSB) as a significant contributor to the ensuing cell death as it cleaves many proteins essential for cell survival. Importantly, chemical inhibition or knockdown of CTSB protects cells from necroptosis. Furthermore, induced polymerization of the MLKL N-terminal domain (NTD) also triggers LMP, leading to CTSB release and subsequent cell death. These findings clearly establish the critical role of MLKL polymerization induced lysosomal membrane permeabilization (MPI-LMP) in the process of necroptosis.

## Introduction

Necroptosis is a regulated form of immunogenic cell death, characterized by organelle swelling, plasma membrane disruption and release of damage-associated molecular patterns [1]. It has been implicated in various human diseases, including inflammation, infection, organ damage and cancer [2–5]. One of the best characterized necroptosis pathways is induced by tumor necrosis factor (TNF). TNF (T) together with a small molecule inhibitor Smac-mimetic [6] (S) and a pan-caspase inhibitor Z-VAD-FMK (Z) induce formation of the necrosome [7]. The core members of the necrosome are receptor-interacting protein kinase 1 and 3 (RIPK1, RIPK3) and mixed lineage kinase-like protein (MLKL) [7–13]. Activated RIPK3 phosphorylates MLKL, leading to the formation of MLKL tetramers, which further polymerize to form disulfide bond-dependent amyloid-like polymers to promote cell death [14]. Although we observed mitochondria fragmentation, nuclear membrane leakage and plasma membrane rupture during necroptosis [15], the involvement of MLKL polymers in these events and the mechanism of cell death remains unclear.

Lysosomes are acidic organelles (pH4.5–5.0) containing many hydrolytic enzymes important for cellular catabolism [16]. Dysregulation of lysosome function often leads to adverse effects on human health. Various insults can trigger lysosomal membrane permeabilization (LMP) which causes the loss of lysosomal membrane integrity and the release of luminal contents into cytosol [17, 18]. LMP often results in cell death that involves lysosomal proteases called cathepsins. Mammalian cells have around 11 different cathpsins [19]; among them, cathepsin B, D and L (CTSB, CTSD, CTSL) are most abundant and are known to be involved in LMP-induced cell death [17, 18].

Our study demonstrates that upon induction of necroptosis, activated MLKL translocates to and polymerizes on the lysosomal membrane. MLKL polymerization-induced LMP (MPI-LMP), causes the release of mature cathepsins, including CTSB. CTSB then cleaves essential proteins to promote cell death. Importantly, our findings reveal that chemical inhibition or knockdown of CTSB can protect cells from necroptosis. Overall, our study provides crucial insights into how MLKL polymers mediate the execution of necroptosis.

## Results

### Lysosomal membrane permeabilization precedes plasma membrane rupture

**Fig. 1:**
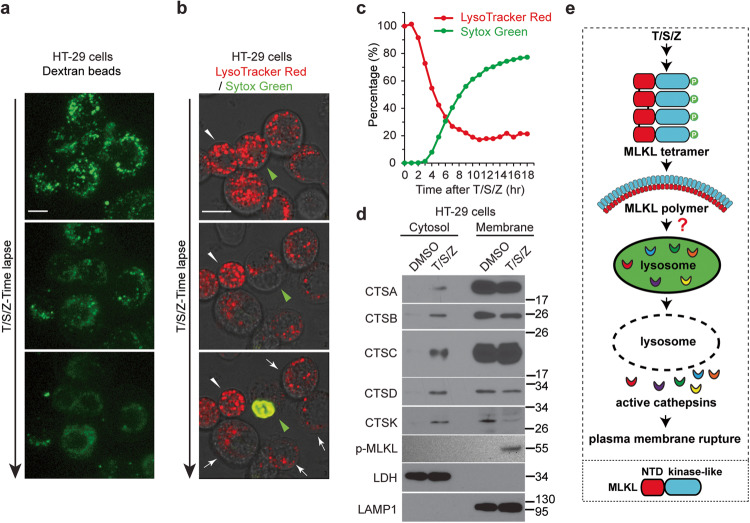
Lysosomal membrane permeabilization precedes plasma membrane rupture and active cathepsins are released into cytosol during necroptosis. **a** HT-29 cells were preloaded with 10 kDa Green Dextran beads overnight. Live cell imaging was recorded after treatment with TNF (T), Smac-mimetic (S) and Z-VAD-FMK (Z). Scale bar, 10 μm. **b** HT-29 cells were stained with 1 μM LysoTracker Red DND-99 for 2 h followed by 3 washes with PBS. Cells were then treated with 1 μM Sytox Green and T/S/Z, followed by live cell imaging. Scale bar, 10 μm. The green arrowhead identifies a cell undergoing necroptosis and the white arrowhead marks a relatively normal neighboring cell. **c** HT-29 cells were seeded at a density of 2000 cells/well and stained with LysoTracker Red and Sytox Green. The cells were then treated with DMSO or T/S/Z and florescent images were captured every hour for 18 h. Percentage of red and green signal intensity at each time point was calculated as described in the methods. **d** HT-29 cells were fractionated into cytosol and membrane fractions after DMSO or T/S/Z treatment for 4 h, followed by Western blotting with the indicated antibodies. The mature active cathepsins were shown. LDH (lactate dehydrogenase) and LAMP1 served as cytosol and membrane markers respectively. Antibody p-MLKL recognizes phospho-S358 of MLKL. **e** Working model. Upon activation, phosphorylated MLKL forms tetramers and later polymers, somehow leading to LMP and the release of active cathepsins into cytosol, followed by plasma membrane rupture. Diagram on the bottom shows two domains of MLKL, the N-terminal domain (NTD) and the C-terminal kinase-like domain.

LMP has been linked to various forms of cell death [17, 18]. It is reported that LMP occurs before plasma membrane rupture in mouse fibrosarcoma L929 cells after necroptosis induction [20]. To investigate if LMP is involved in necroptosis of human cells, 10 kDa Green Dextran beads were preloaded into human colon cancer HT-29 cells. These beads were engulfed by the endocytic pathway and confined into lysosomes showing as green puncta. Upon induction of necroptosis with T/S/Z, green signals gradually disappeared from the lysosome puncta and diffused into the cytosol in almost all cells, indicating LMP (Fig. 1a and Movie S1). We then aimed to determine if LMP occurred before or after plasma membrane damage. To achieve this, we stained cells with LysoTracker Red and incubated them with plasma membrane-impermeable DNA dye Sytox Green. Upon T/S/Z treatment, in the cell marked by the green arrowhead, red lysosome puncta gradually decreased in intensity and number until barely visible, accompanied by eventual plasma membrane rupture, followed by green nuclear staining (Fig. 1b and Movie S2). Notably, adjacent cells (indicated by the white arrow) exhibited a significant decrease in red lysosome staining without green nuclear staining, indicating the occurrence of LMP prior to membrane disruption. In contrast, a neighboring cell with relatively no change in red signal indicates no photobleaching occurred (white arrowhead). In addition, the population analysis revealed a strong negative correlation between LysoTracker Red signal and Sytox Green signal (Fig. 1c). For instance, at 4 h, LysoTracker already dropped to about 55%, while only 8% cells had Sytox Green signal. These results provide strong evidence that LMP occurs prior to plasma membrane rupture during necroptosis in human cells.

### Active cathepsins are released from lysosome lumen into cytosol during necroptosis

The lysosome plays a crucial role in the degradation and recycling of cellular material, which involves various enzymes, including proteases called cathepsins. Cathepsins are synthesized as inactive proenzymes, which are activated by autocleavage or other proteases into smaller mature forms in the acidic late endosomes and lysosomes [19]. To examine whether LMP leads to the release of these proteases, we separated DMSO or T/S/Z-treated HT-29 cells into cytosolic and membrane fractions. Consistent with previous reports, phosphorylated MLKL was detected in the membrane fractions after T/S/Z treatment [7, 14, 21, 22] (Fig. 1d). Notably, mature cathepsins including CTSA, CTSB, CTSC, CTSD and CTSK were released from membrane fraction into cytosol only upon T/S/Z treatment (lane 2, Fig. 1d). Together, our results demonstrate that LMP occurs during necroptosis, leading to the release of lysosomal proteases and plasma membrane rupture (Fig. 1e).

### Activated MLKL translocates to the lysosomal membrane after necroptotic induction

**Fig. 2:**
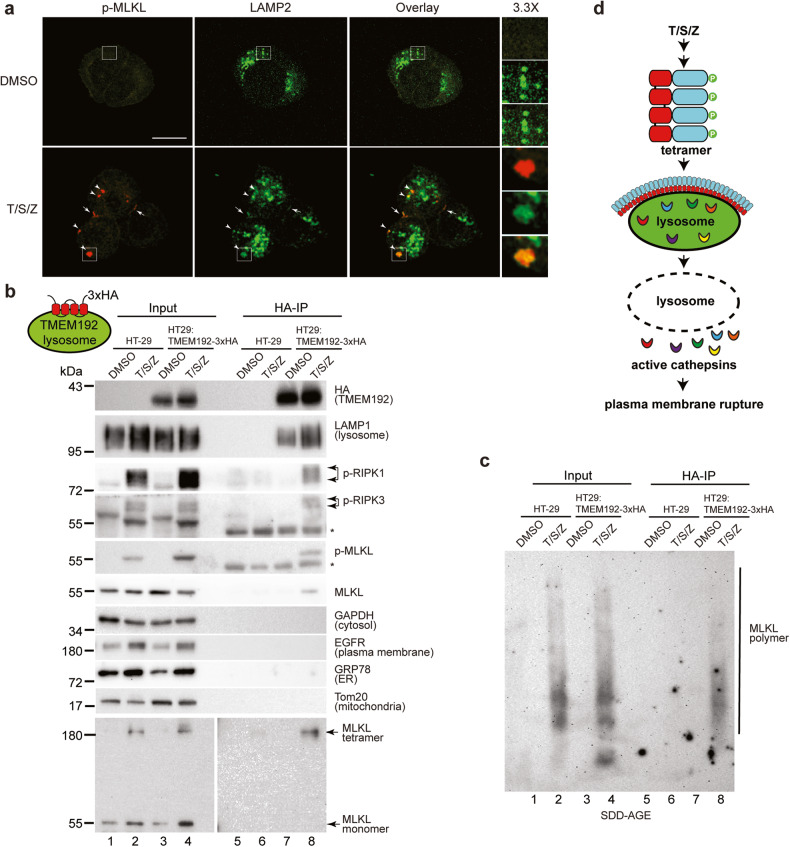
Activated MLKL translocates to the lysosomal membrane after necroptotic induction. **a** HT-29 cells were treated with DMSO or T/S/Z for 4 h, followed by staining with antibodies against p-MLKL and LAMP2. Arrowheads mark cytosolic puncta and arrows denote plasma membrane puncta. **b** Lysosomal membrane protein TMEM192 with 3×HA tags at C-terminus was stably expressed in HT-29 cells to establish HT-29:TMEM192-3xHA cell line. Cells were treated with DMSO or T/S/Z for 4 h and lysosomes were precipitated with anti-HA magnetic beads as described in methods. Western blotting was performed with the indicated antibodies. * denotes non-specific signals from the IgG heavy chain. For tetramer detection, non-reducing SDS-PAGE was performed. LAMP1, lysosome marker; GAPDH, cytosol marker; EGFR, plasma membrane marker; GRP78, ER marker; and Tom20, mitochondria marker. Antibodies against phospho-S166 of RIPK1 (p-RIPK1) and phospho-S227 of RIPK3 (p-RIPK3) were also used. **c** Immunoprecipitated lysosomes were subjected to semi-denaturing detergent agarose gel electrophoresis (SDD-AGE) and Western blotting was performed with an MLKL antibody. **d** Working model. Upon activation, MLKL translocates to the lysosome membrane, leading to LMP and release of active cathepsins into cytosol, and eventual plasma membrane rupture.

Next, we wanted to investigate the possible role of MLKL in LMP. MLKL has been reported to translocate to plasma membrane [21–24] and organelle membranes (including mitochondria, ER and lysosome) [25, 26] during necroptosis. To this end, phospho-S358-MLKL antibody was used to visualize MLKL activation and translocation [7]. Very little phospho-MLKL was present in DMSO-treated HT-29 cells. Upon T/S/Z treatment, phospho-MLKL formed large puncta in the cytosol (arrowheads in bottom left panel, Fig. 2a and Fig. S1a) and on the cell membrane including cell-cell contact membrane (arrows in bottom left panel, Fig. 2a). Importantly, most puncta co-stained with lysosomal membrane protein LAMP2, suggesting that activated MLKL translocates to lysosomal membrane (middle and right panel, Fig. 2a). Furthermore, some of the phospho-MLKL puncta colocalized with large areas of cytosolic LAMP2 staining which might represent clusters of lysosomes (inset, Fig. 2a).

Sabatini’s group developed a lysosome-immunoprecipitation (IP) protocol to purify lysosomes from cells [27]. Similarly, we stably expressed 3xHA-tagged lysosomal membrane protein TMEM192 in HT-29 cells. The immunoprecipitated lysosomes were largely free of cytosol, plasma membrane, ER and mitochondria (Fig. 2b). Notably, upon T/S/Z treatment, core necrosome components, including phospho-RIPK1, phospho-RIPK3 as well as phospho-MLKL specifically co-precipitated with lysosomes (lane 8, Fig. 2b). We and others have reported that phospho-MLKL oligomerizes to form disulfide bond-linked tetramers [21, 22, 25], which then polymerize to produce amyloid-like polymers [14]. Importantly, MLKL tetramers and polymers co-precipitated with purified lysosomes (lane 8, Fig. 2b, c). Additionally, we performed Optiprep density gradient centrifugation to isolate lysosomes from HeLa:GFP-RIPK3:MLKL cells, which stably express 3xFlag-tagged MLKL [14]. Similar to the lysosome-IP results, MLKL tetramers and polymers were found to co-segregate with fractions enriched in the lysosomes of T/S/Z-treated cells (Fig. S1b–d) These results suggest that the necrosome translocates to the lysosomal membrane, where phosphorylated MLKL forms tetramers and polymers which might in turn promote cell death (Fig. 2d).

### MLKL polymerizes on the lysosomal membrane to promote lysosome fusion and lysosomal membrane permeabilization

**Fig. 3:**
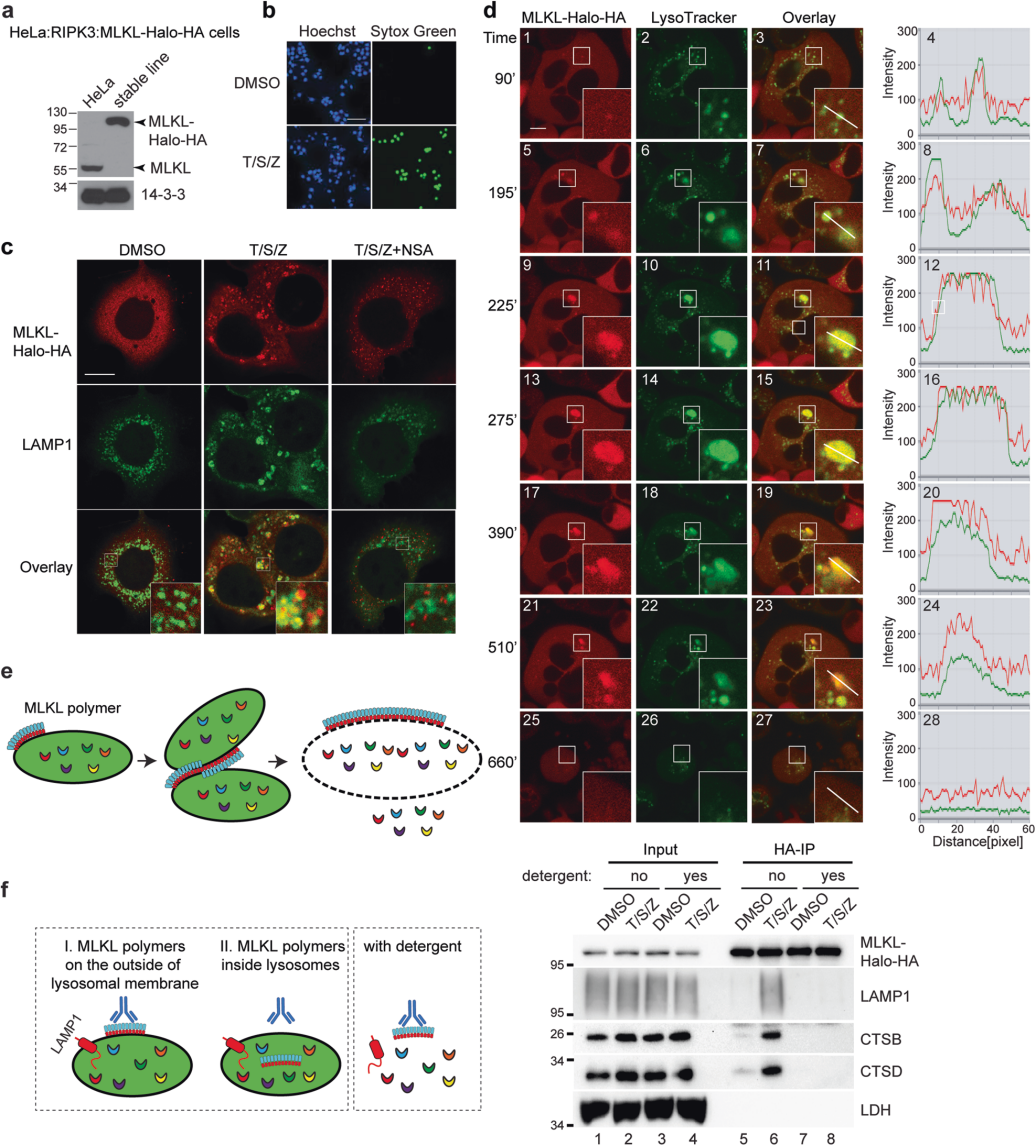
Activated MLKL polymerizes on the lysosomal membrane to promote lysosome fusion and lysosomal membrane permeabilization. **a** Characterization of the HeLa:RIPK3:MLKL-Halo-HA cell line. After CRISPR/Cas9-mediated knockout of endogenous MLKL in HeLa cells, MLKL fused with C-terminal Halo-Tag and HA-tag as well as FLAG-RIPK3 was engineered to stably express in these cells. Western blotting was performed with an MLKL antibody. **b** HeLa:RIPK3:MLKL-Halo-HA cells were treated with DMSO or T/S/Z overnight and then stained with Hoechst and a cell-impermeable DNA dye Sytox Green. Hoechst stains all cells and Sytox Green stains dead cells with compromised cell membranes. Scale bar, 100 μm. **c** Cells were treated with the indicated inducers for 4 h, and the fluorescent dye TMR was added to stain MLKL-Halo. Cells were then fixed and stained with an anti-LAMP1 antibody. Insets were shown at a 4 × magnification. Scale bar: 10 μm. **d** HeLa:RIPK3:MLKL-Halo-HA cells were stained with TMR for MLKL-Halo and LysoTracker Green DND-26 for lysosomes. Live cell imaging was recorded after T/S/Z treatment. Insets were shown at a 3 × magnification. A line intensity profile of the inset was analyzed with Zeiss software ZEN and shown on the right. Scale bar: 10 μm. **e** Working model. Upon activation, MLKL polymerizes on the lysosomal membrane, and MLKL polymers on different lysosomes further polymerize to promote lysosome clustering and fusion, eventually leading to LMP. **f** The left panel depicts a diagram illustrating the predicted outcomes of MLKL-Halo-HA-IP based on its localization, either outside or inside lysosomes. HeLa:RIPK3:MLKL-Halo-HA cells were treated with DMSO or T/S/Z for 4 h and harvested as described for lysosome-IP. MLKL-Halo-HA fusion protein was precipitated with anti-HA magnetic beads with or without 1% Triton X-100. Western blotting was performed with the indicated antibodies.

To visualize the movement of MLKL in real-time, we stably expressed MLKL-Halo-HA fusion protein in HeLa cells with endogenous MLKL inactivated by CRISPR/Cas9 and stably expressed RIPK3 (Fig. 3a). These cells readily underwent necroptosis with T/S/Z treatment (Fig. 3b). Similar to HT-29 cells, inhibition of RIPK1 with necrostatin-1 (Nec-1) or inhibition of MLKL with necrosulfonamide (NSA) blocks necroptosis in these cells (Fig. S2). The fluorescent dye TMR covalently conjugates to HaloTag and labels the fusion protein, which colocalized with the lysosomal membrane protein LAMP1 upon T/S/Z treatment (Fig. 3c). Interestingly, large MLKL-Halo puncta co-stained with clusters of lysosomes (inset, Fig. 3c), suggesting that MLKL polymerization might nucleate lysosome clustering. The MLKL inhibitor necrosulfonamide (NSA) which inhibits MLKL polymerization [14] was found to block MLKL colocalization with the lysosome, suggesting a possible link between lysosome translocation and MLKL polymerization.

In the live-cell recordings, dispersed MLKL initially formed small red dots on individual lysosomes with strong lysotracker green staining (panel 1–4, Fig. 3d; Movie S3). These MLKL-Halo positive lysosomes then began to converge and fuse to form larger vesicles, and the large vesicles continued to attract and fuse with small lysosomes that also showed MLKL-Halo signal (panel 5–16, Fig. 3d). Initially, these large, fused lysosomes showed strong red and green signals. However, as the lysosomes reached their maximal size, the green lysotracker signal began to decrease until undetectable, indicating the occurrence of LMP (panel 17–28, Fig. 3d). These results suggest that MLKL polymers on the lysosomal membrane may interact with polymers on other lysosomes to promote lysosome clustering and fusion. The continued growth of MLKL polymers on the fused large lysosomes eventually lead to LMP (Fig. 3e).

MLKL, along with other necrosome components, has been reported to undergo degradation inside lysosomes under various conditions [28–30]. In order to determine whether MLKL is localized on the outside of lysosomal membrane or inside lysosomes during necroptosis, we performed an anti-HA antibody IP to isolate tagged MLKL and its associated materials from HeLa:RIPK3:MLKL-Halo-HA cells. The results shown in Fig. 3f demonstrated a strong interaction between MLKL and lysosomes, which was only observed after T/S/Z treatment. This interaction was confirmed by the presence of lysosomal membrane protein LAMP1 and lysosome luminal enzymes CTSB and CTSD (lane 6). Conversely, when lysosome membrane was disrupted with detergent, MLKL did not associated with lysosomal markers (lane 8), indicating that MLKL’s interaction with lysosomes relies on the lysosomal membrane. These results suggest that at least a portion of MLKL localizes on the outside of lysosomal membrane during necroptosis and the interaction is robust enough to co-precipitate with lysosomes. It is important to note that our results do not exclude the possibility that other portions of MLKL and necrosome components may still undergo degradation inside lysosomes.

### Inhibition of lysosomal cysteine protease CTSB attenuates necroptosis

**Fig. 4:**
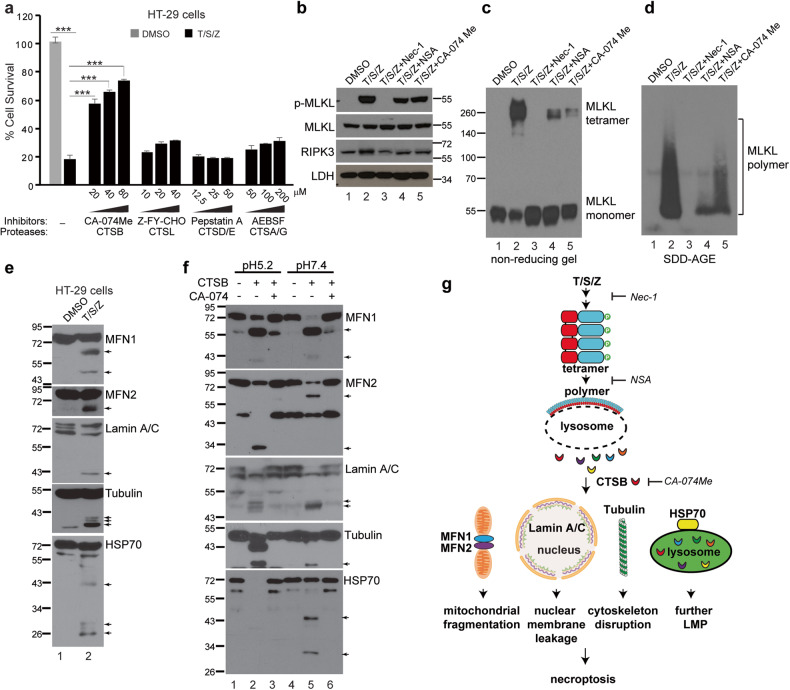
Inhibition of lysosomal cysteine protease CTSB attenuates necroptosis and CTSB cleaves vital proteins at neutral pH. **a** HT-29 cells were treated with DMSO or T/S/Z for 16 h in the presence of various concentrations of different protease inhibitors and cell survival was assayed by CellTiter-Glo. ***p < 0.001, mean ± SD are shown. **b** HT-29 cells were treated with the indicated inducers for 4 h and cell lysates were subjected to Western blotting with the indicated antibodies. 20 μM of CA-074Me, 10 μM of Nec-1 (RIPK1 inhibitor) and 5 μM of NSA (MLKL inhibitor) were used. Cell lysates were analyzed by non-reducing SDS-PAGE (**c**) or SDD-AGE (**d**) and probed with an MLKL antibody. **e** HT-29 cells were treated with DMSO or T/S/Z for 4 h and cell lysates were subjected to Western blotting with the indicated antibodies. Arrows denote the cleaved bands. **f** In vitro CTSB cleavage assay. Recombinant Tubulin or HSP70, or membrane fractions from HT-29 cells which were used as starting material for MFN1, MFN2, and Lamin A/C, were incubated with 100 ng of recombinant CTSB under conditions of pH5.2 or pH7.4, followed by Western blotting. CA-074 (20 μΜ) is a CTSB inhibitor. Arrows denote the cleaved bands. **g** Working model. Upon activation, MLKL tetramers form polymers on the lysosome membrane, leading to LMP and the release of active cathepsins into cytosol. Released CTSB cleaves MFN1, MFN2, Lamin A/C, Tubulin and HSP70 to promote mitochondrial fragmentation, nuclear membrane leakage, cytoskeleton disruption and further lysosome permeabilization, eventually resulting in cell death.

LMP triggers the release of active cathepsins (Fig. 1c). To investigate the functional significance of these proteases, a panel of cathepsin inhibitors were added to T/S/Z-treated HT-29 cells. The results demonstrated that the CTSB inhibitor CA-074Me provided strong protection against necroptosis, whereas the CTSL inhibitor Z-FY-CHO and CTSA/G inhibitor AEBSF only provided weak protection, and the CTSD/E inhibitor pepstatin A provided no protection (Fig. 4a). These findings suggested that CTSB plays a crucial role in necroptosis. Furthermore, Z-VAD-FMK which inhibits cysteine proteases caspases, did not affect the activity of cysteine protease CTSB under our treatment condition (Fig. S3a).

Necroptosis induction triggers MLKL phosphorylation, which leads to the formation of tetramers and eventually polymers [14]. To investigate at which step the CTSB inhibitor impedes cell death, HT-29 cells were treated with T/S/Z and either RIPK1 inhibitor Nec-1, MLKL inhibitor NSA, or CA-074Me. Non-reducing gel electrophoresis was performed to detect MLKL tetramers and semi-denaturing detergent agarose gel electrophoresis (SDD-AGE) was performed to detect MLKL polymers. As reported before [13, 14], Nec-1 blocked RIPK1 activity and prevented MLKL phosphorylation, tetramer and polymer formation (lanes 2 and 3 in Fig. 4b–d). NSA blocked MLKL polymer formation, but MLKL phosphorylation and tetramer formation still took place albeit to a lesser extent than treatment with T/S/Z alone (lane 4 in Fig. 4b–d). Interestingly, in CA-074Me treated cells, MLKL phosphorylation, tetramer and polymer formation still occurred, also to a lesser extent than T/S/Z treatment alone (lane 5 in Fig. 4b–d).

### CTSB cleaves proteins vital for cell survival at cytosolic neutral pH

A sequence of morphological changes occurs during necroptosis, including mitochondrial fragmentation, nuclear membrane breakage, and plasma membrane rupture [15]. To investigate the proteins involved in these events, Western blotting was performed, and it was found that two mitochondria fusion proteins (MFN1 and MFN2), nuclear membrane structural proteins (Lamin A/C), a cytoskeleton protein (Tubulin) and a chaperone protein (HSP70) were cleaved upon necroptosis induction (Fig. 4e).

CTSB is a lysosomal protease which normally functions at acidic pH. However, recent studies have shown that CTSB can perform proteolytic cleavage at a neutral pH once released from lysosomes [31, 32]. To investigate the effect of CTSB on key proteins involved in necroptosis, recombinant CTSB was incubated with membrane fractions or purified recombinant Tubulin or HSP70 under pH5.2 or pH7.4 conditions in vitro. At pH5.2, CTSB readily cleaved MFN1, MFN2, Lamin A/C, Tubulin and HSP70 and this proteolytic activity was inhibited by CA-074, a cell-impermeable version of CA-074Me (lanes 2 and 3, Fig. 4f). Notably, CTSB was also able to cleave these proteins at pH7.4, and the cleavage pattern closely resembled what was observed in T/S/Z-treated cells, while at pH5.2 the cleavage was often more complete (Fig. 4e, f). These results suggest that released CTSB after MPI-LMP cleaves essential proteins in the neutral cytosol, which contributes to mitochondrial fragmentation, nuclear membrane leakage and disruption of the cytoskeleton. In addition, HSP70 is a crucial stabilizer of lysosomes [33], and the loss of HSP70 protection will destabilize more lysosomes, resulting in excessive LMP (Fig. 4g) This process establishes a positive feedback loop, amplifying the detrimental effects of the assault.

### Loss of CTSB suppresses protein cleavage and necroptosis

**Fig. 5:**
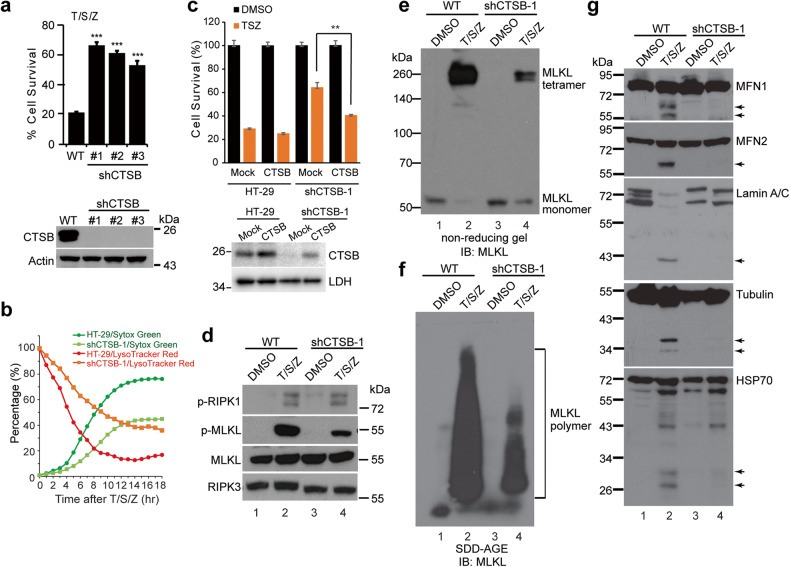
Loss of CTSB suppresses protein cleavage and necroptosis in HT-29 cells. **a** Upper panel, parental HT-29 (WT) and CTSB knockdown (shCTSB) cells were treated with DMSO or T/S/Z for 16 h. Cell survival was measured by CellTiter-Glo assay. ***p < 0.001. Lower panel, Western blotting with antibodies against CTSB and Actin. **b** LysoTracker Red and Sytox Green staining images for HT-29 or shCTSB-1 cells were recorded and analyzed as in Fig. 1c. **c** Upper panel, HT-29 or shCTSB-1 cells were transfected with an empty vector or a CTSB expressing plasmid that is resistant to shRNA. Thirty-six hours later, the cells were treated with T/S/Z for 16 h and CellTiter-Glo was performed to assay cell survival. Lower panel, Western blotting with antibodies against CTSB and LDH. **d** Cells were treated with DMSO or T/S/Z for 4 h and cell lysates were subjected to Western blotting with the indicated antibodies. Cell lysates were analyzed with non-reducing SDS-PAGE (**e**) or SDD-AGE (**f**) and probed with an MLKL antibody. **g** Cell lysates were subjected to Western blotting with the indicated antibodies. Arrows denote cleaved bands.

Further investigations were conducted to explore the function of CTSB by performing loss of function experiments in HT-29 cells. Stable knockdown of CTSB with shRNA led to strong protection against necroptosis in multiple clones, similar to the inhibition observed with CA-074Me (Fig. 5a). Notably, shCTSB cells exhibited slower and lesser reduction in lysoTracker signal compared to HT-29 cells, corresponding to lesser increase in Sytox Green signal (Fig. 5b). Moreover, when CTSB was reintroduced into shCTSB cells, cell death was rescued (Fig. 5c). In addition to shRNA-mediated knockdown, CRISPR/Cas9-mediated stable knockout of CTSB also inhibited necroptosis, further supporting the essential role of CTSB in the necroptotic pathway (Fig. S3b, c). At the molecular level, RIPK1-ser166 phosphorylation still occured in shCTSB cells upon T/S/Z-treatment, suggesting that upstream signal is not affected (Fig. 5d). Furthermore, in shCTSB cells, MLKL phosphorylation, MLKL tetramer formation and polymer formation still took place (Fig. 5e, f). Moreover, cleavage of MFN1, MFN2, Lamin A/C, Tubulin and HSP70 was blocked in CTSB knockdown cells (Fig. 5g), supporting the important role of CTSB in the degradation of essential proteins to promote necroptosis. It is important to note that the levels of p-MLKL, MLKL tetramers and polymers were reduced in shCTSB cells compared to WT cells. This decrease could be at least partly attributed to the disruption of the positive feedback loop of LMP caused by the loss of CTSB function. Alternatively, it might suggest that CTSB plays additional roles in promoting MLKL activation.

### NTD-DmrB forms polymers on the lysosomal membrane and induces LMP during necroptosis

We previously established an NTD-DmrB cell line which stably expressed the N-terminal domain (NTD) of MLKL fused to a dimerization domain (DmrB) in HeLa cells with endogenous MLKL inactivated by CRISPR-Cas9 [14]. These cells readily undergo necroptosis with dimerizer (D) treatment, and we commonly include Z-VAD-FMK (Z) to ensure that apoptosis does not occur. Since NTD-DmrB cells do not need RIPK1, RIPK3 and endogenous MLKL to activate necroptosis, it is interesting to examine if LMP performs a similar role in cell death execution in these cells.

**Fig. 6:**
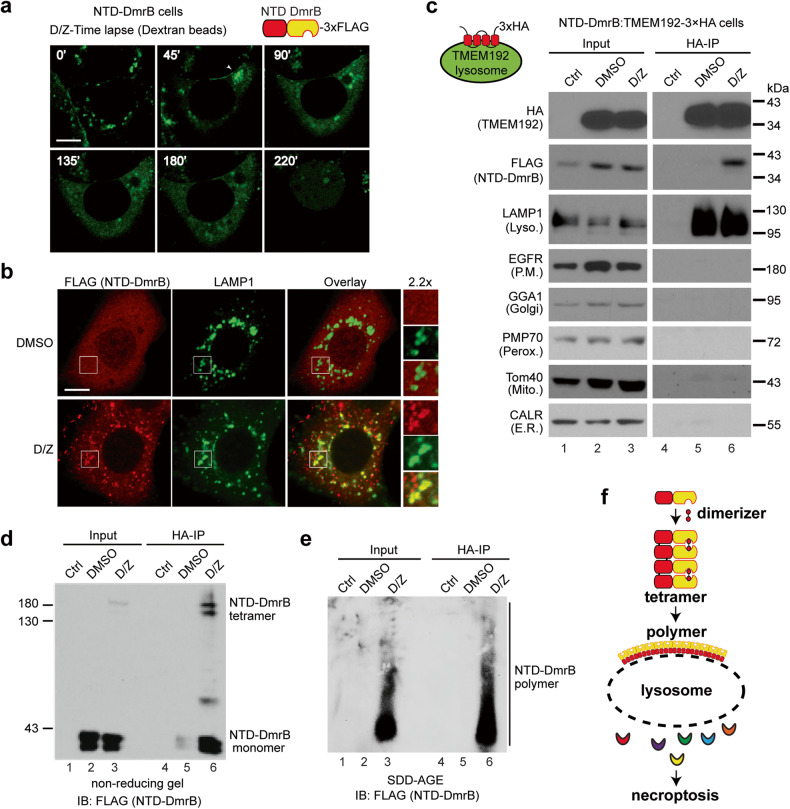
NTD-DmrB forms polymers on the lysosomal membrane and induces LMP during necroptosis. **a** NTD-DmrB cells were preloaded with green Dextran beads and live cell imaging was recorded following dimerizer (D) and Z-VAD-FMK (Z) treatment. Scale bar, 10 μm. **b** NTD-DmrB cells were treated with DMSO or D/Z for 2 h and stained with antibodies against FLAG and LAMP1. Scale bar, 10 μm. **c** Lysosomal membrane protein TMEM192 with 3×HA tags at C-terminus was stably expressed in NTD-DmrB cells. Cells were treated with DMSO or D/Z for 2 h and lysosomes were precipitated with anti-HA magnetic beads and Western blotting was performed with the indicated antibodies. Control sample was from DMSO-treated parental NTD-DmrB cells. LAMP1, lysosome marker; EGFR, plasma membrane marker; GGA1, Golgi marker; PMP70, peroxisome marker; Tom40, mitochondria marker; CALR (Calreticulin), ER marker. Immunoprecitated lysosomes were analyzed with non-reducing SDS-PAGE (**d**) or SDD-AGE (**e**) and probed with an anti-FLAG antibody. **f** Working model. Upon D/Z treatment, NTD-DmrB tetramers form polymers on lysosomal membrane and induces LMP.

In NTD-DmrB cells, Green Dextran beads were engulfed and stored in lysosomes, displaying as distinct puncta. Upon D/Z treatment, some of the puncta began to cluster (arrowhead, Fig. 6a). Subsequently, numerous puncta disappeared, and the green signals diffused into the cytosol, indicating LMP (Fig. 6a). Immunostaining revealed that NTD-DmrB protein was typically dispersed in the cytosol. However, upon D/Z treatment, it formed puncta, many of which colocalized with the lysosome marker LAMP1 (Figs. 6b and S4a). Notably, some of the larger LAMP1 puncta resembled lysosome clusters (inset, Fig. 6b). These findings suggest that activated NTD-DmrB also translocates to lysosomes to promote LMP.

To confirm the translocation of NTD-DmrB to lysosomes and its role in promoting LMP, lysosome-IP was conducted in NTD-DmrB cells that stably expressed TMEM192-3xHA. The results indicated that NTD-DmrB tetramers and polymers were present exclusively on isolated lysosomes following D/Z treatment (lane 6, Fig. 6c–e). Similar outcomes were obtained through Optiprep density gradient centrifugation (Fig. S4b–d). These findings provide further evidence that NTD-DmrB migrates to lysosomes and polymerizes on the lysosomal membrane to stimulate LMP, leading to cell death (Fig. 6f).

### Loss of CTSB prevents protein cleavage and suppresses necroptosis in NTD-DmrB cells

**Fig. 7:**
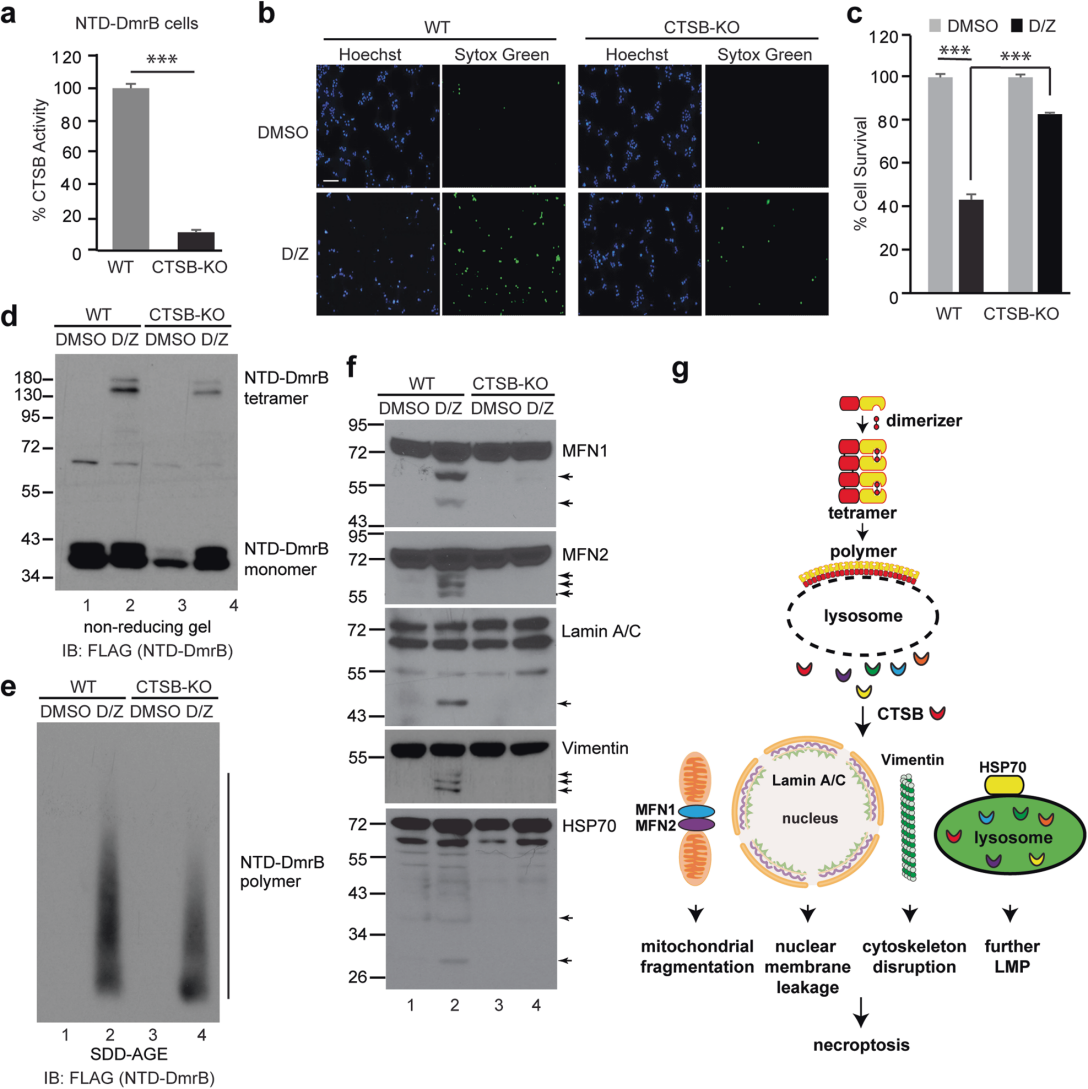
Loss of CTSB prevents proteins cleavage and suppresses necroptosis in NTD-DmrB cells. **a** CTSB was inactivated by CRISPR/Cas9-mediated knockout in NTD-DmrB cells and CTSB enzyme activity was analyzed in lysates from WT and CTSB-KO cells. ***p < 0.001. **b** Cells were treated with DMSO or D/Z for 8 h and stained with Hoechst and Sytox Green. Scale bar, 100 μm. **c** Cells were treated with DMSO or D/Z for 8 h and cell survival was analyzed by CellTiter-Glo assay. ***p < 0.001. Cells were treated for 2 h and cell lysates were analyzed by non-reducing SDS-PAGE (**d**) or SDD-AGE (**e**) and probed with a FLAG antibody. **f** Cell lysates were analyzed by Western blotting with the indicated antibodies. Arrows denote cleaved bands. **g** Working model. Upon D/Z treatment, NTD-DmrB tetramers form polymers on the lysosomal membrane, leading to LMP and release of active cathepsins into cytosol. Released CTSB cleaves MFN1, MFN2, Lamin A/C, Vimentin and HSP70 to promote mitochondrial fragmentation, nuclear membrane leakage, cytoskeleton disruption and further lysosome permeabilization, eventually resulting in cell death.

CTSB was inactivated in NTD-DmrB cells by CRISPR/Cas9, confirmed by a CTSB activity assay (Fig. 7a). In these cells, necroptosis was suppressed, measured by Sytox Green staining and CellTiter-Glo assay (Fig. 7b, c). Furthermore, when CTSB was reintroduced into CTSB-KO cells, cell death was restored (Fig. S5a, b), confirming the vital role of CTSB in NTD-DmrB-mediated necroptosis. Similar to what was observed in HT-29 cells, NTD-DmrB tetramers and polymers still formed in CTSB-KO cells (Fig. 7d, e). Importantly, cleavage of MFN1, MFN2, Lamin A/C, cytoskeleton protein Vimentin as well as HSP70 was suppressed in CTSB-KO cells (Fig. 7f). Taken together, these results suggest that necroptosis execution in NTD-DmrB cells is similar to that observed in HT-29 cells, involving NTD-DmrB polymerization-induced LMP, and CTSB release, which in turn cleaves many vital proteins to promote cell death (Fig. 7g).

## Discussion

In this study, we discovered that MPI-LMP leads to the release of lysosomal proteases, including CTSB, which in turn cleaves numerous essential proteins, contributing to the eventual demise of the cell. Upon induction of necroptosis, phosphorylated MLKL forms polymers on the lysosomal membrane, as confirmed through immuno-staining, lysosome-IP, and Optiprep density gradient experiments (Figs. 2 and S1). Live cell imaging revealed that MLKL polymers on the lysosomal membrane continue to recruit and polymerize with other MLKL polymers, resulting in lysosome clustering and fusion, ultimately resulting in permeabilization of the large, fused lysosomes (Fig. 3 and Movie S3). Moreover, NTD-DmrB exhibits similar translocation and polymerization activity on the lysosomal membranes, which also leads to LMP (Fig. 6).

The exact mechanism by which MLKL polymers permeabilize lysosomes is currently unknown. One possibility is that MLKL polymers stiffen local membrane domains, resulting in uneven membrane tension. As the fused lysosomes grow larger, the uneven tension becomes stronger and eventually leads to membrane rupture. A similar mechanism has been proposed for human islet amyloid polypeptide protein which forms amyloid fibrils to directly disrupt vesicle membranes and promote the death of insulin-producing islet beta cells [34]. Another model suggests that positively charged residues in the N-terminal domain of MLKL interact directly with negatively charged phospholipids in the membrane, disrupting membrane integrity [23–25, 35]. Alternatively, MLKL oligomers might form a pore [36, 37] or channel as suggested before [38–40], inducing membrane permeabilization. Finally, MLKL polymers might recruit other membrane effector proteins that contribute to membrane disruption [21]. These possibilities are not mutually exclusive, and it is possible that MLKL polymers may concentrate a large number of positively charged residues at a local membrane domain, assisting the amyloid-like fibers in disrupting the membrane.

Necrosome and subsequent MLKL oligomers/polymers have been detected on various membrane compartments. The plasma membrane is a commonly reported site for MLKL translocation, as evidenced by several studies [20–23]. However, other studies have reported MLKL translocation to organelles such as mitochondria, ER, lysosomes or autolysosomes [25, 26, 41], as well as the nucleus [42]. Additionally, MLKL has been observed on endocytic and exocytic vesicles [43, 44]. Recently, a study demonstrated that MLKL forms clusters on cytosolic vesicles before translocating to hotspots on the plasma membrane, particularly at cell-cell junctions [45]. These observations suggest that MLKL translocation may be a multi-step process and that the formation of MLKL polymers on lysosomal membranes may represent a crucial step in this process.

It has been reported that TNF binding to TNFR1 triggers endocytosis of the receptor with its associated complex I [46], leading to necrosome nucleation on vesicles that contain caveolin-1-associated detergent-resistant membrane [47]. Inhibition of MLKL with NSA results in colocalization of RIPK3 on caveolin-1 containing vesicles [47], consistent with our earlier report that necrosomes and MLKL tetramers are present in membrane fractions after cotreatment with T/S/Z and NSA [14]. However, under this condition, MLKL polymers do not form, and cell death does not occur. In combination with our observation that NSA results in smaller MLKL puncta which do not colocalize with lysosomes (Fig. 3c), it suggests that NSA might inhibit the process of MLKL translocation from caveolin-1 vesicles to lysosomes, and that MLKL tetramers need to translocate to the lysosomal membrane to efficiently polymerize. It is possible that there are lysosome-specific proteins or membrane domains that are necessary for MLKL recruitment and polymerization. It is reported that lysosomal Rag-Ragulator Complex recruits RIPK1 and Caspase 8 to mediate Yersinia-induced pyroptosis [48]. It will be interesting to examine if the same complex is involved in lysosome recruitment of MLKL. Moreover, lysosomes are part of the intracellular membrane system which exchanges membrane components with the plasma membrane. It is plausible that MLKL polymers are transported to the plasma membrane through lysosome membrane exchange, leading to disruption of the plasma membrane. In summary, we propose that MLKL translocation involves multiple steps. Upon T/S/Z treatment, clathrin-mediated endocytosis internalizes complex I, which initiates necrosome formation on caveolin-1 containing vesicles. These vesicles then transport necrosomes to various organelles, particularly lysosomes. On the lysosomal membrane, MLKL undergoes polymerization to induce clustering and fusion of lysosome, eventually resulting in LMP. Additionally, MLKL polymers may also be transported to the plasma membrane through membrane exchange mediated by lysosomes or vesicles, ultimately leading to plasma membrane rupture. Future experiments are crucial for elucidating the distinct functions of MLKL within various cellular compartments in the regulation of necroptosis.

Our proposed mechanism diverges from the previously suggested role for the lysosome in breaking down necrosome components, such as phospho-MLKL [28–30, 49]. Moreover, it is distinct from endocytic and exocytic events that inhibit MLKL function and cell death [43, 44]. Importantly, these processes do not trigger LMP.

We believe that lysosome proteases play an active and important role in the execution of necroptosis. MPI-LMP results in the rapid release of mature cathepsins into cytosol, where they degrade many proteins essential for lysosome stability, mitochondrial fusion, nuclear membrane integrity as well as cytoskeletal structure, ultimately leading to extensive cellular damage and death. While cathepsins typically function at low pH, growing evidence suggests that they exhibit significant activity at neutral pH [18]. For instance, the release of CTSB into the cytosol was shown to promote cell death in acute pancreatitis [32]. Indeed, at pH7.4, recombinant CTSB is able to cleave MNF1/2, Lamin A/C, Tubulin and HSP70, with a pattern similar to what is observed in necroptotic cells (Fig. 4f). Additional mass spectrometry experiments are necessary to find out what other proteins are cleaved during necroptosis. Critically, chemical inhibition of CTSB as well as CTSB knockdown or knockout protects cells from necroptosis, confirming its crucial role in necroptosis execution (Figs. 4, 5 and 7). Interestingly, in cells with diminished CTSB function, reduced levels of phospho-MLKL, MLKL tetramers and polymers are detected (Figs. 4 and 5). One potential explanation is that loss of CTSB disrupts the positive feedback loop of LMP, leading to a reduction in further lysosomal damage. This, in turn, could contribute to a dampening effect on MLKL activation. An alternative explanation is that CTSB might have additional roles in promoting MLKL activation. Previous reports have indicated that cathepsins, including CTSB, are involved in the proteolytic cleavage of endosomal proteins such as Toll-like receptor 3, 7 and 9 on the luminal side, which is crucial for their proper functioning [50]. A plausible speculation is that CTSB might perform a similar cleavage function for a protein located on the lysosomal membrane that is crucial for MLKL activation. Future experiments are required to investigate these possibilities.

The specific cathepsin(s) involved is not obvious since there are about 11 different cathepsins in human cells. Inhibitor experiments suggest that CTSL, CTSD/E and CTSA/G may not have major roles in this type of cell death in HT-29 cells; however, we cannot rule out the contribution of other cathepsins since 30% of cells still die upon CTSB inhibition. Additionally, other degradation enzymes in the lysosome, such as lipases, glycosidase and nucleases, may also contribute to membrane damage and cell death. It is also conceivable that different cell types may utilize different cathepsins to carry out cell death execution since cathepsins can be differentially expressed [19].

LMP has been previously linked with many types of cell death, including apoptosis, necrosis or pyroptosis [17, 18]. It is proposed that the extent of lysosome damage might dictate what types of cell death it induces. For instance, limited damage triggers apoptosis while more extensive assault can lead to necrosis [17]. However, what distinguishes MPI-LMP so that it activates necroptosis instead of other types of cell death? The answer may lie in the fact that MLKL polymerization promotes lysosome clustering and fusion, resulting in the formation of large lysosomes (Fig. 3c, d). When these large lysosomes are permeabilized by MLKL polymers, they release a massive amount of cathepsins, overwhelming the neutralizing capacity of cytosolic inhibitors of cathepsins such as stefins and serpins [17]. Furthermore, the release of a massive number of protons from these large lysosomes may lead to acidification of the cytosol, enhancing cathepsin activity. Additionally, released CTSB cleaves HSP70, further damaging the remaining lysosomes to amplify the assault, leading to an uncontrolled protease rampage. These findings highlight the crucial role played by MPI-LMP in the execution of necroptosis and provide valuable insights for developing lysosome integrity-based therapeutic strategies to alleviate necroptosis-associated diseases.

## Methods

### Cell culture, antibodies and reagents

All cells were cultured in DMEM with high glucose and 10% fetal bovine serum and 1% penicillin/streptomycin. For Tet-on system, 100 ng/ml Doxycycline was supplemented to induce target gene expression. The following antibodies were purchased from Santa Cruz: anti-CTSA (sc-26049), anti-CTSB (sc-6493 and sc-13985), anti-CTSC (sc-74590), anti-CTSD (sc-53927), anti-CTSK (sc-6506), anti-MFN1 (sc-100561), anti-MFN2 (sc-100562), anti-Vimentin (sc-73259), anti-Lamin A/C (sc-6215), anti-GGA1 (sc-30102), anti-14-3-3 (sc-629), anti-Tom40 (sc-11414), anti-LAMP1 (sc-17768). And the following antibodies were from Abcam: anti-p-Ser-358 MLKL (ab187091), anti-RIPK3 (phospho-S227) (ab209384), anti-LDH (ab53292) and anti-PMP70 (ab-3421). The following antibodies were from Cell Signaling: anti-phospho-RIP (Ser166) (#65746), anti-phospho-RIPK3 (Ser227) (#93654), anti-EGFR (#2232), anti-HA-HRP (#14031), anti-Calreticulin (#2891) and anti-Tubulin (#2146). Other antibodies include, anti-MLKL (GTX107538, GeneTex), anti-RIPK1 (610459, BD), anti-Flag M2-HRP (A8592, Sigma), anti-LAMP2 (abcam, ab25631) and anti-HSP70 (ADI-SPA-812-F, Enzo). Anti-RIPK3, Smac-mimetic and recombinant TNFα was generated as described before [6, 51]. Z-VAD-FMK (ZVAD), CA-074 Me, CA-074, Pepstatin A and AEBSF were purchased from ApexBio. The following compounds were also purchased, including Necrostatin-1 (Nec-1, Calbiochem), Necrosulfonamide (NSA, Millipore), Z-FY-CHO (EMD Millipore), and Dimerizer (635058, Clontech).

### Cell death assays

Cell death assays were described previously [52]. Briefly, 2000 cells were seeded in each well of the 96-well plate with Dox if needed. On the second day, cells were treated with different necroptotic inducers in triplicate with or without inhibitorsfor indicated times. CellTiter-Glo assays were carried out according to the manufacturer’s instructions (Promega) and luminescent data were recorded with *Synergy*^TM^ 2 Multi-Mode Microplate Reader from BioTek. The percentage of cell survival was calculated as (luminescence signal in inducer-treated wells/luminescence signal in DMSO-treated wells) *100. For imaging, 1 μM of Sytox Green (S7020, ThermoFisher) and 100 μg/ml Hoechst (H3570, ThermoFisher) were applied. The images were taken with a Cytation 3 imaging reader from BioTek. Except otherwise specified, 20 ng/ml TNFα, 100 nM Smac-mimetic and 20 μM Z-VAD-FMK were used to induce cell death in HT-29 cells. For NTD-DmrB cells, 20 nM dimerizer and 20 μM Z-VAD-FMK were used.

### Cell fractionation and western blotting

To fractionate cells into cytosol and membrane fractions, cells were scraped and harvested followed by three washes with ice-cold PBS. The cell pellets were resuspended into Buffer A (20 mM Tris-HCl, pH7.4, 10 mM KCl, 1.5 mM MgCl_2_) with protease inhibitors and phosphatase inhibitors and sat on ice for 30 min. The swollen cells were passed through G22 needle for 25 strokes and spun down at 500 g for 10 min. The supernatants were collected by sequential centrifugations at 20,000 g for 12 min and at 100,000 g for 1 h. The resulting supernatants (S100) were used as cytosol fractions. The pellets from 20,000 g spin were washed with Buffer A and extracted by Lysis Buffer (50 mM Tris-HCl, pH7.4, 137 mM NaCl, 1% Triton X-100, 10% Glycerol) as membrane fractions extract (P20 extract). For whole cell extracts, cells were harvested as above and lysed in the Lysis Buffer with protease inhibitors and phosphatase inhibitors for 30 min on ice. After centrifugation at 12,000 g, the supernatants were collected. Protein concentration was measured with Pierce Protein Assay Kit (#23225 ThermoFisher). Protein extracts were prepared in 1×SDS sample buffer (50 mM Tris-HCl, pH6.8, 2% SDS, 10% Glycerol, 2.5% β-mercaptoethanol and 0.05% Bromophenol Blue) and boiled for 5–10 min for regular Western blotting. To examine tetramer formation, whole cell extracts were prepared in 1×SDS sample buffer without β-mercaptoethanol (β-ME). Proteins were resolved in SDS-PAGE gel and transferred to PVDF membrane.

### Semi-Denaturating Detergent Agarose Gel Electrophoresis (SDD-AGE)

SDD-AGE was done as previously described [53]. Briefly, 100 μg of whole cell extracts was prepared in 1× SDD-AGE sample buffer (0.5 × TAE, 5% glycerol, 2% SDS, and 0.02% bromophenol blue) and loaded in 1% horizontal Agarose gel containing 0.1% SDS. The gel was run at 4 V/cm gel length in 1 × TAE buffer with 0.1% SDS for 4–5 h. The proteins were transferred onto PVDF membrane by capillary action in TBS buffer (20 mM Tris, pH 7.4, 150 mM NaCl). The resulting membrane was then subjected to Western blotting.

### CRISPR/Cas9 mediated knockout and shRNA techniques

All the CRISPR/Cas9 mediated knockout cell lines were generated according to the protocol described before [54, 55]. Briefly, the viral particles were produced by transfecting HEK293T cells with the viral gRNA vector containing target sequence and packaging vectors pMD2.G, and psPAX2. The viruses were harvested at 48 h and 72 h post-transfection and filtered. The pre-seeded recipient cells were infected by virus with 6 µg/ml Polybrene. One day after infection, the recipient cells were divided into 15 cm dishes with the corresponding antibiotic media for selection. Eventually single clones were selected and expanded. HT-29-shCTSB stable cell line was established as described before [15]. After antibiotic selection, survival cells were expanded. Gene knockdown or knockout was confirmed by sequencing and western blotting or CTSB activity assay. The following target sequences were used: MLKL-KO, GCTGCCCTGGAGGAGGCTAATGG, as described before [14]; CTSB-KO, TCAACAAACGGAATACCACG; shCTSB, GCACCGATCAGTACTGGGA.

### CTSB activity assay

(1) CTSB activity measurement: whole cell extract was prepared using 0.2% Triton Lysis Buffer (50 mM Tris-HCl, pH7.4, 137 mM NaCl, 0.2% Triton X-100, 10% Glycerol) without protease inhibitors and phosphatase inhibitors. 10–20 µg of whole cell extract was incubated with 10 µM of CTSB substrate Z-RR-AMC and 1 mM DTT in 1× cathepsin assay buffer (50 mM NaAC, pH5.2 and 5 mM EDTA) for 1 h at 30 °C. Fluorescence intensity was read with a *Synergy*™ *2* Multi-Mode Microplate Reader from BioTek in 384 well plate at excitation 360/40 and emission 460/40. (2) In vitro CTSB activity assay at pH5.2 and pH7.4. The substrates, including 40 µg of cell membrane fraction P20 for MFN1/MFN2 and LaminA/C, 1.5 µg of commercial Tubulin protein (HTS02-A, Cytoskeleton), or 250 ng of homemade recombinant HSP70, were diluted in 1× cathepsin assay buffer (50 mM NaAC, pH5.2 and 5 mM EDTA) or in Buffer A (20 mM Tris-HCl, pH7.4, 10 mM KCl, 1.5 mM MgCl2) with 10 mM DTT. Purified CTSB protein (BML-SE198-0025, Enzo) was pre-incubated at 37 °C for 5 min for self-activation and 100 ng was then added to the digestion reaction with or without 20 µM CA-074. All reactions were performed at 37 °C for 1 h.

### Immunofluorescence and live cell imaging

For immunofluorescence, cells were plated on cover glass in a 24-well plate. Four hours after DMSO, T/S/Z or T/S/Z/NSA treatment, cells were fixed and stained as described before [56]. TMR (G8251, Promega) was supplemented as instructed by manufacturer prior to fixation. All images were taken with Nikon Super-Resolution microscope. For live cell imaging, cells were plated into 35 mm petri dish with glass bottom (P35G-1.5-14-C, MatTek). The following dyes were used according to manufactures’ instructions, Dextran Beads (D1821, ThermoFisher), LysoTracker Red DND-99 (L7528, Thermofisher), LysoTracker Green DND-26 (L7526, Thermofisher), and Sytox Green (S7020, Thermofisher). After adding T/S/Z or D/Z to the plates, cell death was monitored with Nikon A1R microscope.

### LysoTracker Red and Sytox Green time course

Two thousand cells were seeded in each well of a 96-well plate. On the second day, cells were treated with 1 μM of LysoTracker Red DND-99 (L7528, Thermofisher) for 2 h. Afterwards, the medium was replaced with fresh medium supplemented with 1 μM Sytox Green. The cells were then treated with DMSO, or T/S/Z and florescent images were captured every hour using a Cytation 3 imaging reader for 18 h. The signal percentage at each time point is calculated using the following formulas:

$${{{{{\rm{Percentage}}}}}}\,{{{{{\rm{of}}}}}}\,{{{{{\rm{LysoTracker}}}}}}\,{{{{{\rm{Red}}}}}}= 	 \,({{{{{\rm{Signal}}}}}}\,{{{{{\rm{intensity}}}}}}\,{{{{{\rm{of}}}}}}\,{{{{{\rm{T}}}}}}/{{{{{\rm{S}}}}}}/ {{{{{\rm{Z}}}}}}\,{{{{{\rm{treated}}}}}}\,{{{{{\rm{wells}}}}}})/ \\ 	\,({{{{{\rm{Signal}}}}}}\,{{{{{\rm{intensity}}}}}}\,{{{{{\rm{of}}}}}}\,{{{{{\rm{DMSO}}}}}}\,{{{{{\rm{treated}}}}}}\,{{{{{\rm{wells}}}}}})* 100$$

$$ 	 {{{{{\rm{Percentage}}}}}}\,{{{{{\rm{of}}}}}}\,{{{{{\rm{Sytox}}}}}}\,{{{{{\rm{Green}}}}}}=({{{{{\rm{Signal}}}}}}\,{{{{{\rm{intensity}}}}}}\,{{{{{\rm{of}}}}}}\,{{{{{\rm{T}}}}}}/{{{{{\rm{S}}}}}}/{{{{{\rm{Z}}}}}}\,{{{{{\rm{treated}}}}}}\,{{{{{\rm{wells}}}}}})/ \\ 	({{{{{\rm{Signal}}}}}}\,{{{{{\rm{intensity}}}}}}\,{{{{{\rm{of}}}}}}\,{{{{{\rm{DMSO}}}}}}\,{{{{{\rm{treated}}}}}}\,{{{{{\rm{wells}}}}}}\,{{{{{\rm{lysed}}}}}}\,{{{{{\rm{with}}}}}}\,0.5 \% \,{{{{{\rm{Triton}}}}}}\,{{{{{\rm{X}}}}}}-100)* 100$$

### Rescue experiment

Two thousand HT-29 or shCTSB-1 cells were seeded in each well of a 96 well plate. On the second day, the cells were transfected with 50 ng of either an empty plasmid or a plasmid encoding CTSB with silence mutations that render it resistant to shRNA. Thirty-six hours later, the cells were treated with DMSO or T/S/Z for 16 h, followed by CellTiter-Glo assay. For Western blotting, 5 × 10^4^ cells were seeded in a 24-well plate. On the second day, 500 ng DNA was transfected into the cells.

### Lysosome immunoprecipitation (Lysosome-IP)

Lysosome-IP was carried out as described [27]. Briefly, the cells were scraped, pelleted and resuspended in 1 ml of KPBS (136 mM KCl, 10 mM KH2PO4, pH7.25) with protease inhibitors and phosphatase inhibitors. The suspensions were homogenized by dounce homogenizer. After spinning at 1000 g for 2 min, the supernatants were incubated with the pre-washed HA-magnetic beads (#88837, Pierce) for 30 min. The complexes were washed once with KPBS, twice with KPBS containing 500 mM NaCl and once with KPBS. Finally, the beads were boiled in 1xSDS loading buffer and subjected to Western blotting. SDS sample buffer without β-mercaptoethanol was used for tetramer detection. For SDD-AGE analysis, the beads were eluted with 1xSDD-AGE sampling buffer at 55 °C for 10 min.

### Optiprep density gradient centrifugation

The experiments were carried out according to the instructions of Lysosome Isolation Kit (# LYSISO1, Sigma). Cells were pelleted and resuspended in 1× Exaction buffer and broken by Dounce homogenizer with Pestle B. The samples were then centrifuged at 1000 g for 10 min. The supernatants were further spun at 20,000 g for 20 min. The resulting pellets were resuspended in 1× Exaction buffer by a pellet pestle as Crude Lysosome Fraction (CLF). CLF fractions were then prepared as 19% Diluted OptiPrep Fraction, and the other gradient fractions ranging from 8% to 27% were built according to manufacturer’s instructions. All gradient fractions were sequentially laid in a centrifugation tube and centrifuged at 150,000 g for 5 h. A total of 11 fractions were collected and analyzed by Western blotting, non-reducing SDS-PAGE and SDD-AGE.

### Statistical analysis

Statistical analyses were performed with Excel and GraphPad Prism 8. CellTiter-Glo results are presented as mean +/−SD of *n* = 3 biological independent samples. Two-tailed Student’s *t* test is performed to determine statistical significance. NS, not significant; *P < 0.05; **P < 0.01; ***P < 0.001. Western blotting and immunostaining data are representative of at least three independent experiments with similar results.

### Supplementary information

Supplementary movie legends and figure legends

Lysosomal membrane permeabilization in HT-29 cells during necroptosis.

Lysosomal membrane permeabilization precedes plasma membrane rupture in response to necroptotic stimuli.

Activated MLKL polymerizes on the lysosomal membrane to promote lysosome fusion and lysosomal membrane permeabilization.

MLKL translocates to lysosome fractions upon necroptosis induction.

CellTiter-Glo assay for HeLa:RIPK3:MLKL-Halo-HA cells.

Loss of CTSB suppresses necroptosis.

NTD-DmrB tetramers and polymers are associated with lysosome fractions after cell death induction.

Re-expression of CTSB in CTSB-KO cells rescues cell death.

Original experimental data

checklist

## Data Availability

All the data for the study are included in the published article and the supplementary data files. Additional supporting data are available from the corresponding author upon request.
