# Supplementary material for: MLKL polymerization-induced lysosomal membrane permeabilization promotes necroptosis
Source: Cell Death Differ. 2023 Nov 23;31(1):40–52. doi: 10.1038/s41418-023-01237-7 (PMC10782024; doi:10.1038/s41418-023-01237-7)
Supplement: Supplementary file 1 — Supplementary movie legends and figure legends [file 41418_2023_1237_MOESM1_ESM.docx]

**Supplementary Movies**

Movie S1. (Related to Figure 1a) Lysosomal membrane permeabilization in HT-29 cells during necroptosis. HT-29 cells were preloaded with 10 kDa green Dextran beads overnight. Live cell imaging was recorded with a Nikon Ti microscope with 10 min intervals after treatment with 100 ng/mL TNF (5T), 100 nM Smac-mimetic (S) and 20 μM Z-VAD-FMK (Z). Five times the amount of TNF was used to speed up the process. Imaging analysis was performed with Andor iQ software.

Movie S2. (Related to Figure 1b) Lysosomal membrane permeabilization precedes plasma membrane rupture in response to necroptotic stimuli. HT-29 cells were stained with LysoTracker Red beforehand, incubated with 1 µM Sytox Green and induced with T/S/Z. Live cell imaging was recorded with Nikon A1R microscope with 25 min intervals. Data analysis and Movie were done with NIS-Elements software (Nikon).

Movie S3. (Related to Figure 3d) Activated MLKL polymerizes on the lysosomal membrane to promote lysosome fusion and lysosomal membrane permeabilization. HeLa:RIPK3:MLKL-Halo-HA cells were stained with TMR and green LysoTracker and induced with T/S/Z. Live cell imaging was recorded with Nikon A1R microscope with 15 min intervals. Data analysis and Movie were done with NIS-Elements software (Nikon).

**Supplementary Figures**

Supplementary Figure 1. MLKL translocates to lysosome fractions upon necroptosis induction. (**a**) HT-29 cells were treated with DMSO or T/S/Z for 4 hrs, followed by staining with antibodies against p-MLKL and LAMP2. (b) Isolation of lysosomes by Optiprep density gradient centrifugation was completed according to manufacturer’s instructions (Sigma). Crude lysosomal fractions from DMSO or T/S/Z treated cells were adjusted to 19% (v/v) Optiprep and loaded in the discontinuous density gradients as indicated. After ultracentrifugation, the fractions were isolated at visible interfaces and collected as fractions 1 to 11. (**c**) HeLa:GFP-RIPK3:MLKL cells were treated with DMSO or T/S/Z and subjected to crude lysosome isolation followed by Optiprep Density Gradient Centrifugation. Six out of 11 collected fractions were analyzed by Western blotting, and C-terminal 3xFLAG-tagged MLKL and organelles markers were probed with the indicated antibodies. WCE, whole cell extract; LAMP1, lysosome marker; EGFR, plasma membrane marker; GGA1, Golgi marker; PMP70, peroxisome marker; PGAM5, mitochondria marker; CALR (Calreticulin), E.R. marker. (**d-e**) The collected fractions were subjected to non-reducing SDS-PAGE (**d**) or SDD-AGE analysis (**e**) and probed with a FLAG antibody.

Supplementary Figure 2. CellTiter-Glo assay for HeLa:RIPK3:MLKL-Halo-HA cells. HeLa:RIPK3:MLKL-Halo-HA cells were treated as indicated and cell survival was measured with CellTiter-Glo assay. ***P<0.001.

Supplementary Figure 3. Loss of CTSB suppresses necroptosis. (**a**) Z-VAD-FMK does not inhibit CTSB activity at 20 µM. HT-29 cells were treated with DMSO, 20 µM of Z-VAD-FMK or with 20 µM of CA-074Me for 24h respectively. The cells were washed with ice-cold PBS and lysed with 0.2% Triton Lysis Buffer followed by CTSB activity assay. Thirty-six hours later, cell lysates were harvested, and Western blotting was performed with CTSB and LDH antibodies. (**b**) CTSB was inactivated in HT-29 cells by CRISPR/Cas9. Western blotting was performed with lysates from WT and two knockout clones with antibodies against CTSB and 14-3-3. (**c**) Cells were treated with DMSO or T/S/Z and cell death was measured with CellTiter-Glo. Cell survival after DMSO treatment was set as 100%. ** P<0.01; ***P<0.001.

Supplementary Figure 4. NTD-DmrB tetramers and polymers are associated with lysosome fractions after cell death induction. (**a**) NTD-DmrB cells were treated with DMSO or D/Z for 2 hrs and then stained with antibodies against FLAG and LAMP1. Scale bar, 10 μm. (**b**) NTD-DmrB cells were treated with DMSO or D/Z and subjected to crude lysosome isolation followed by Optiprep Density Gradient Centrifugation. Fractions were analyzed by Western blotting with the indicated antibodies. Whole cell extracts (WCE) were used as a reference. Notably, under DMSO treatment, little NTD-DmrB was found in lysosome fractions, whereas under D/Z treatment, a significant amount of NTD-DmrB translocated into the lysosome fractions. (**c**-**d**) Fractions were analyzed by non-reducing SDS-PAGE (**c**) or SDD-AGE (**d**) and probed with a FLAG antibody.

Supplementary Figure 5. Re-expression of CTSB in CTSB-KO cells rescues cell death. (**a**) NTD-DmrB or CTSB-KO cells were transfected with an empty vector or a CTSB expressing plasmid. Thirty-six hours later, the cells were treated with D/Z for 8 hrs and CellTiter-Glow was performed to assay cell survival. (**b**) NTD-DmrB or CTSB-KO cells were transfected as in (**a**). Thirty-six hours later, cell lysates were harvested, and CTSB activity was measured as described in methods.
