# Supplementary figures and images for: MLKL polymerization-induced lysosomal membrane permeabilization promotes necroptosis
Source: Cell Death Differ. 2023 Nov 23;31(1):40–52. doi: 10.1038/s41418-023-01237-7 (PMC10782024; doi:10.1038/s41418-023-01237-7)

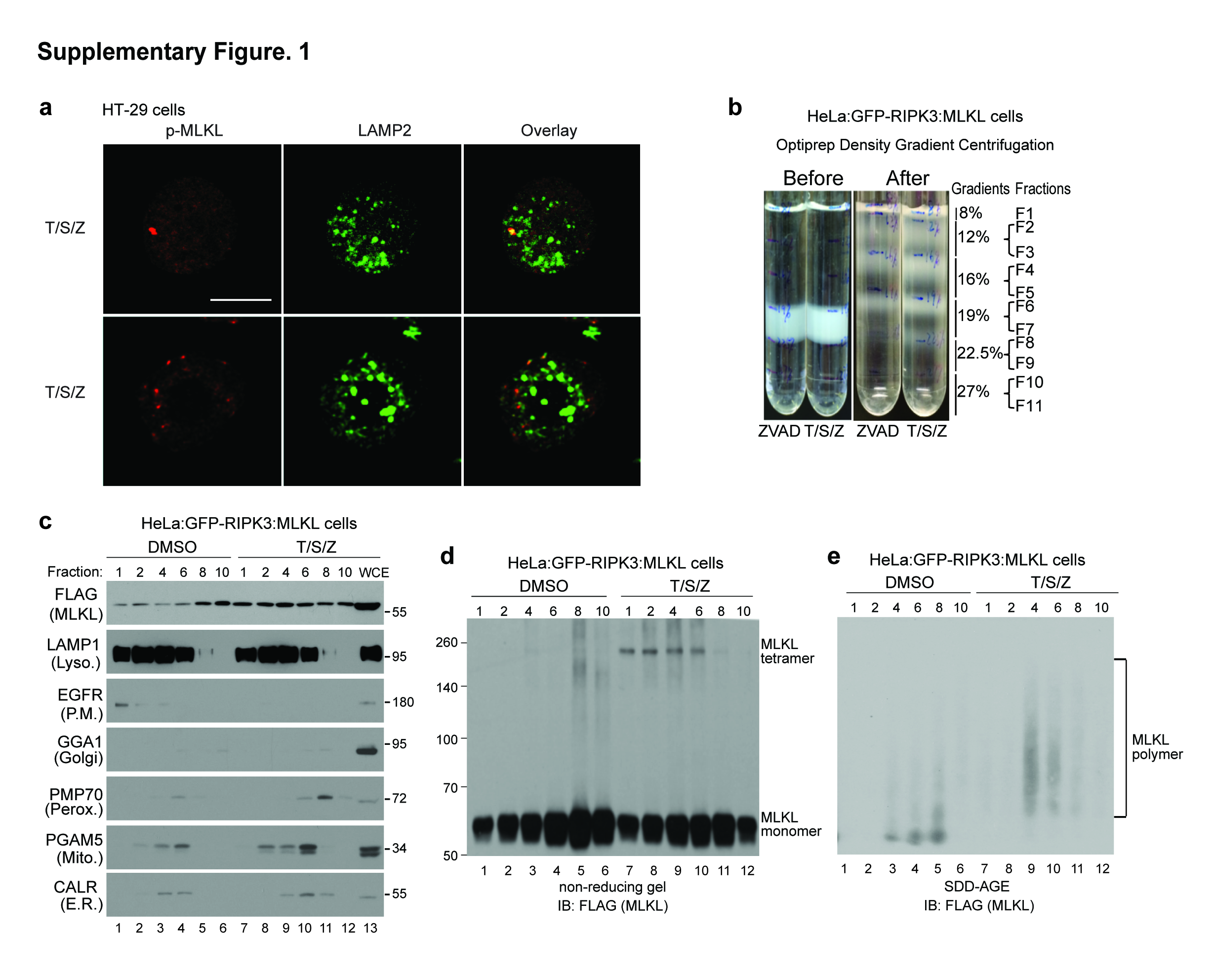

Supplement: Supplementary file 5 — MLKL translocates to lysosome fractions upon necroptosis induction. [file 41418_2023_1237_MOESM5_ESM.tif]

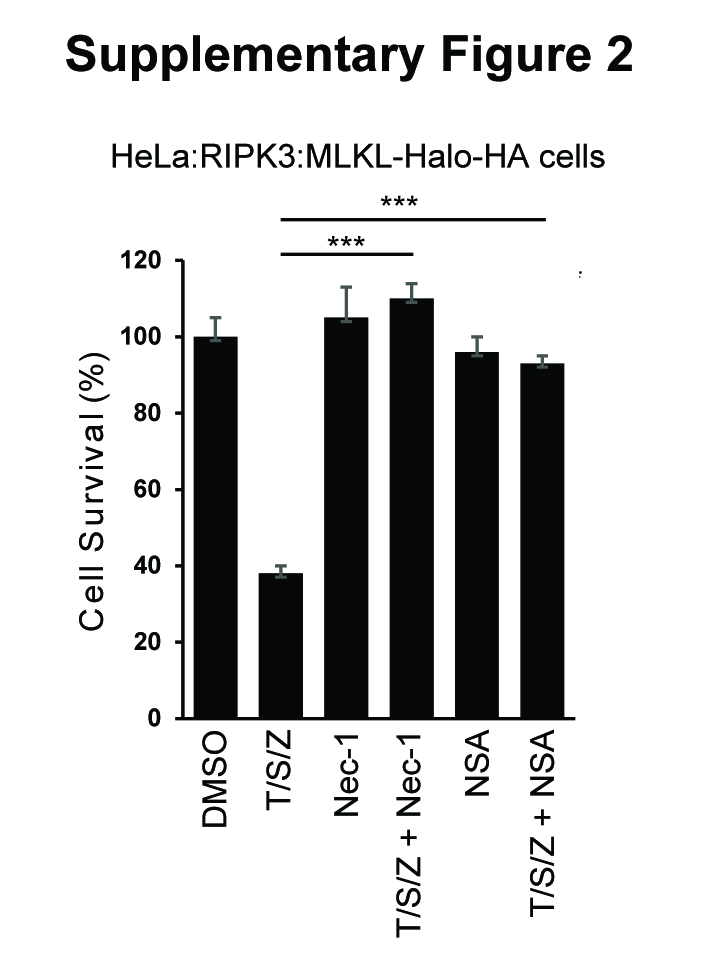

Supplement: Supplementary file 6 — CellTiter-Glo assay for HeLa:RIPK3:MLKL-Halo-HA cells. [file 41418_2023_1237_MOESM6_ESM.tif]

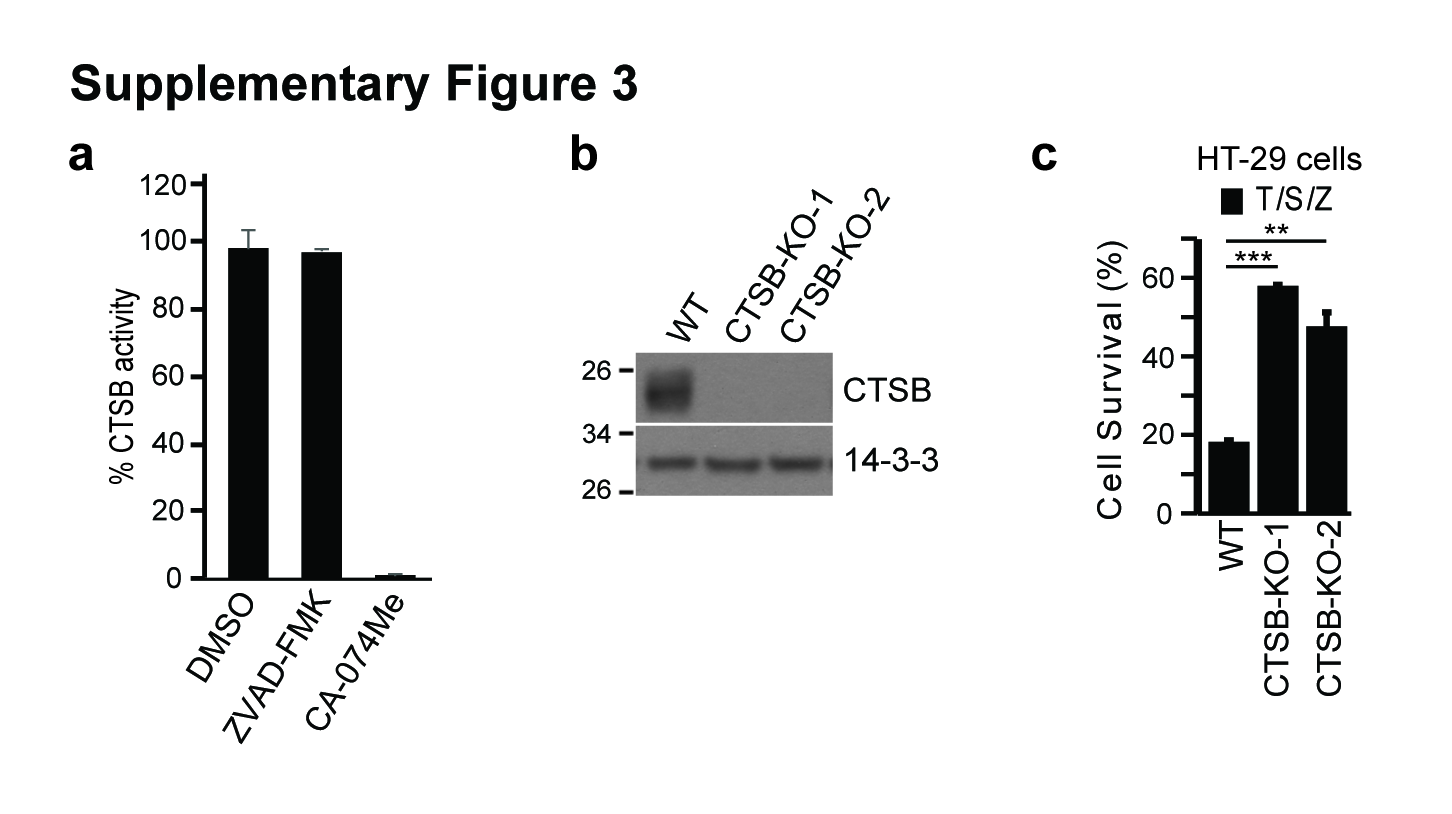

Supplement: Supplementary file 7 — Loss of CTSB suppresses necroptosis. [file 41418_2023_1237_MOESM7_ESM.tif]

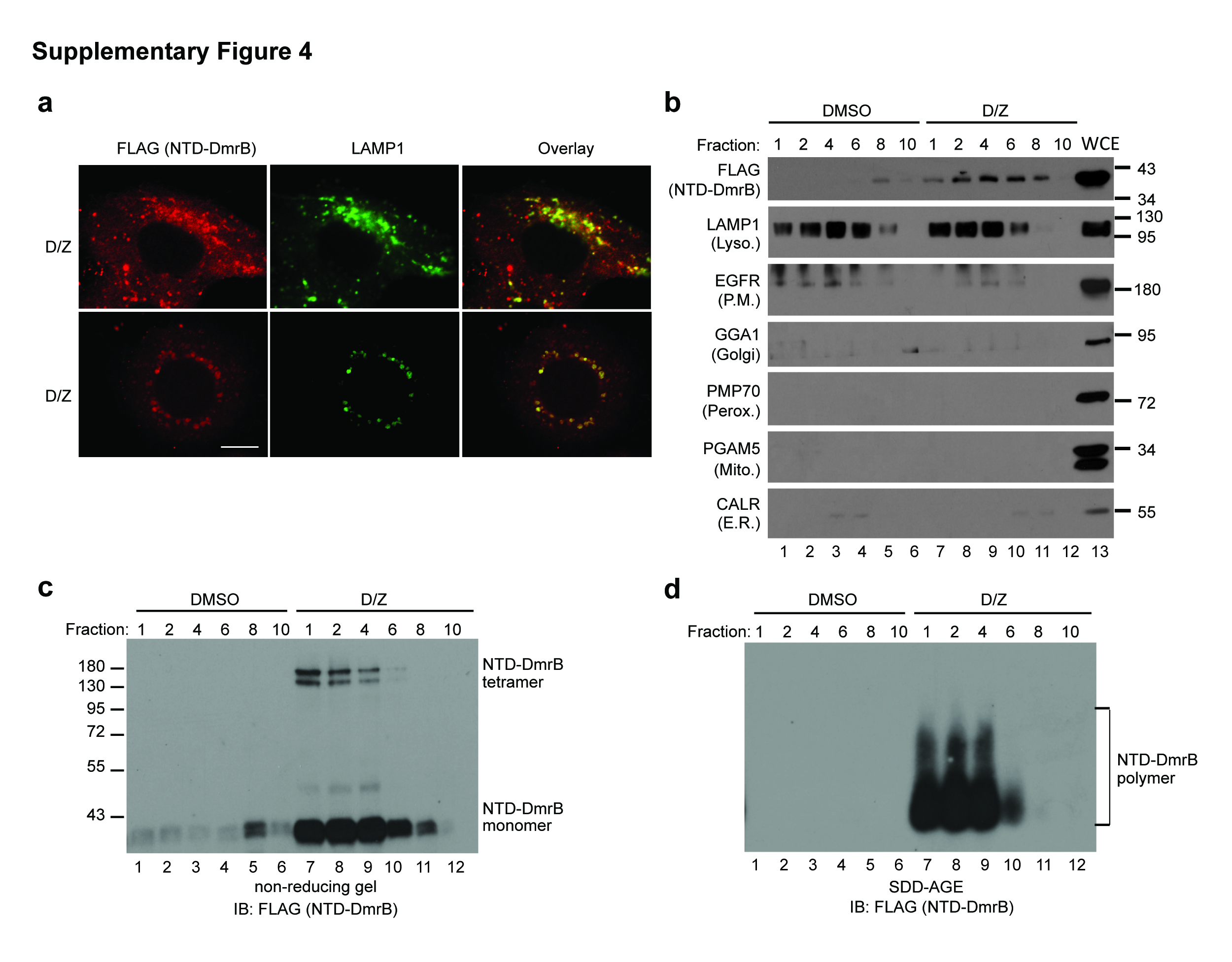

Supplement: Supplementary file 8 — NTD-DmrB tetramers and polymers are associated with lysosome fractions after cell death induction. [file 41418_2023_1237_MOESM8_ESM.tif]

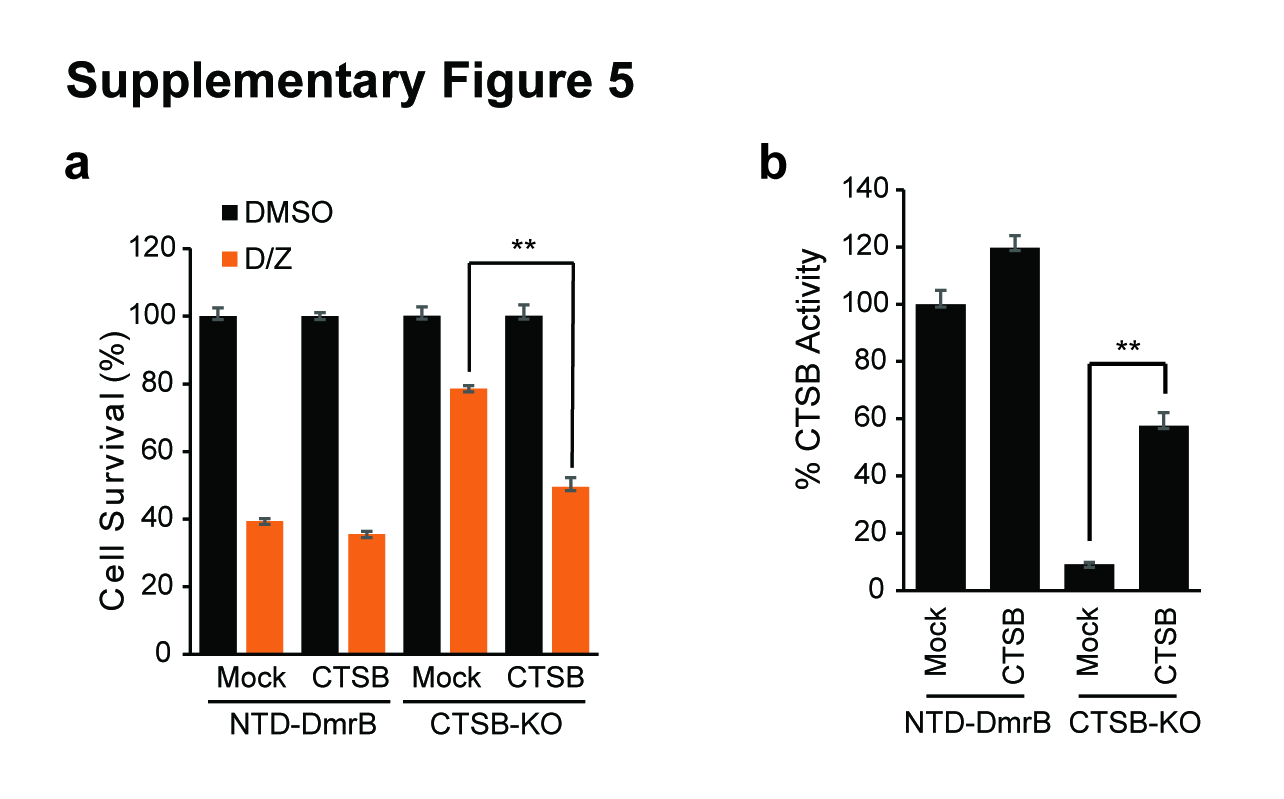

Supplement: Supplementary file 9 — Re-expression of CTSB in CTSB-KO cells rescues cell death. [file 41418_2023_1237_MOESM9_ESM.tif]
