# Supplementary material for: MLKL polymerization-induced lysosomal membrane permeabilization promotes necroptosis
Source: Cell Death Differ. 2023 Nov 23;31(1):40–52. doi: 10.1038/s41418-023-01237-7 (PMC10782024; doi:10.1038/s41418-023-01237-7)

MLKL polymerization-induced lysosomal membrane permeabilization promotes necroptosis

Supplemental Material – Original Blots

Relevant areas for cropped blots in the main and Extended Data figures are shown with red boxes.

Supplemental Material to Fig. 1d (original blots)

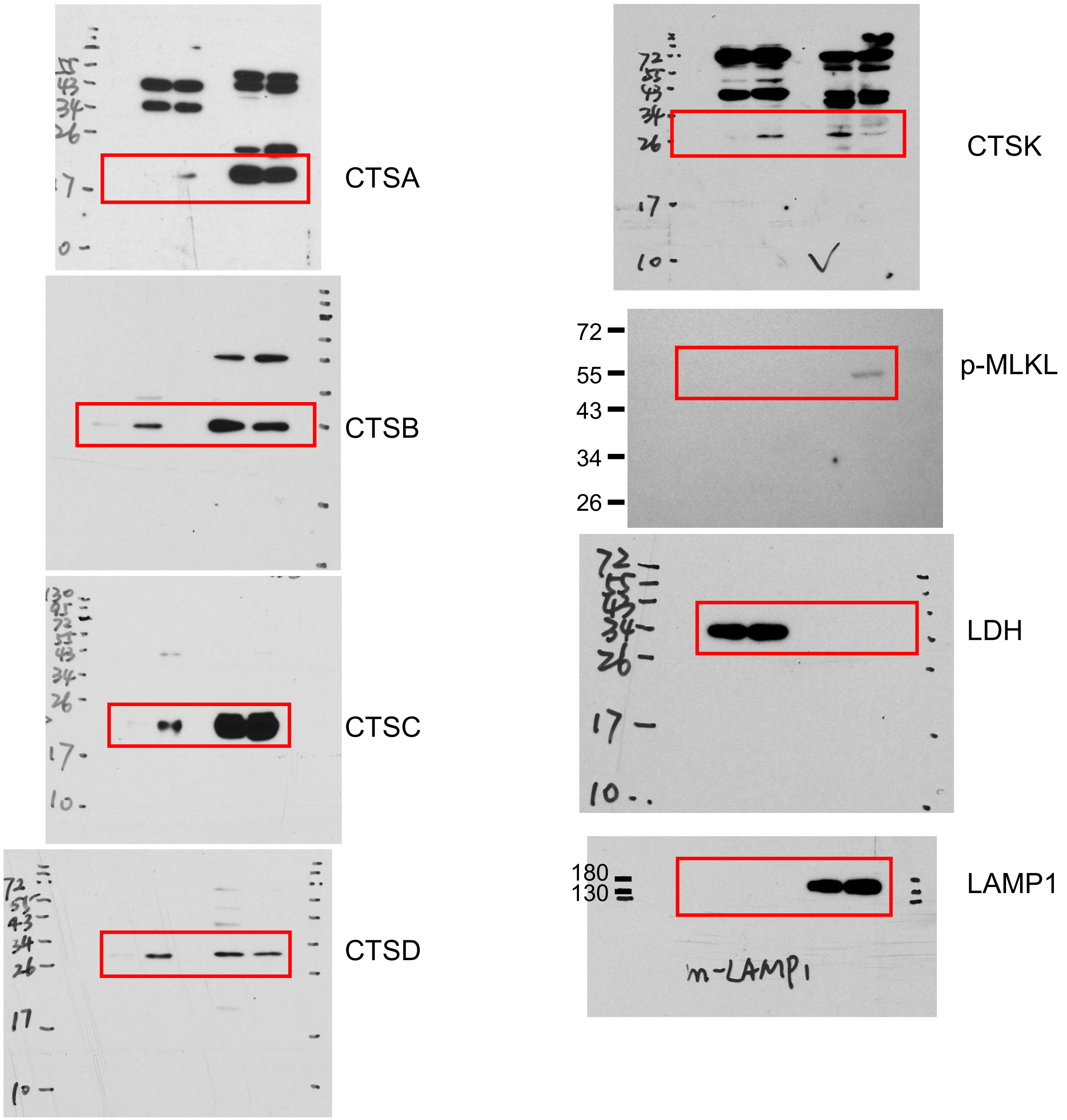

Supplemental Material to Fig. 2 (original blots)

Fig. 2b

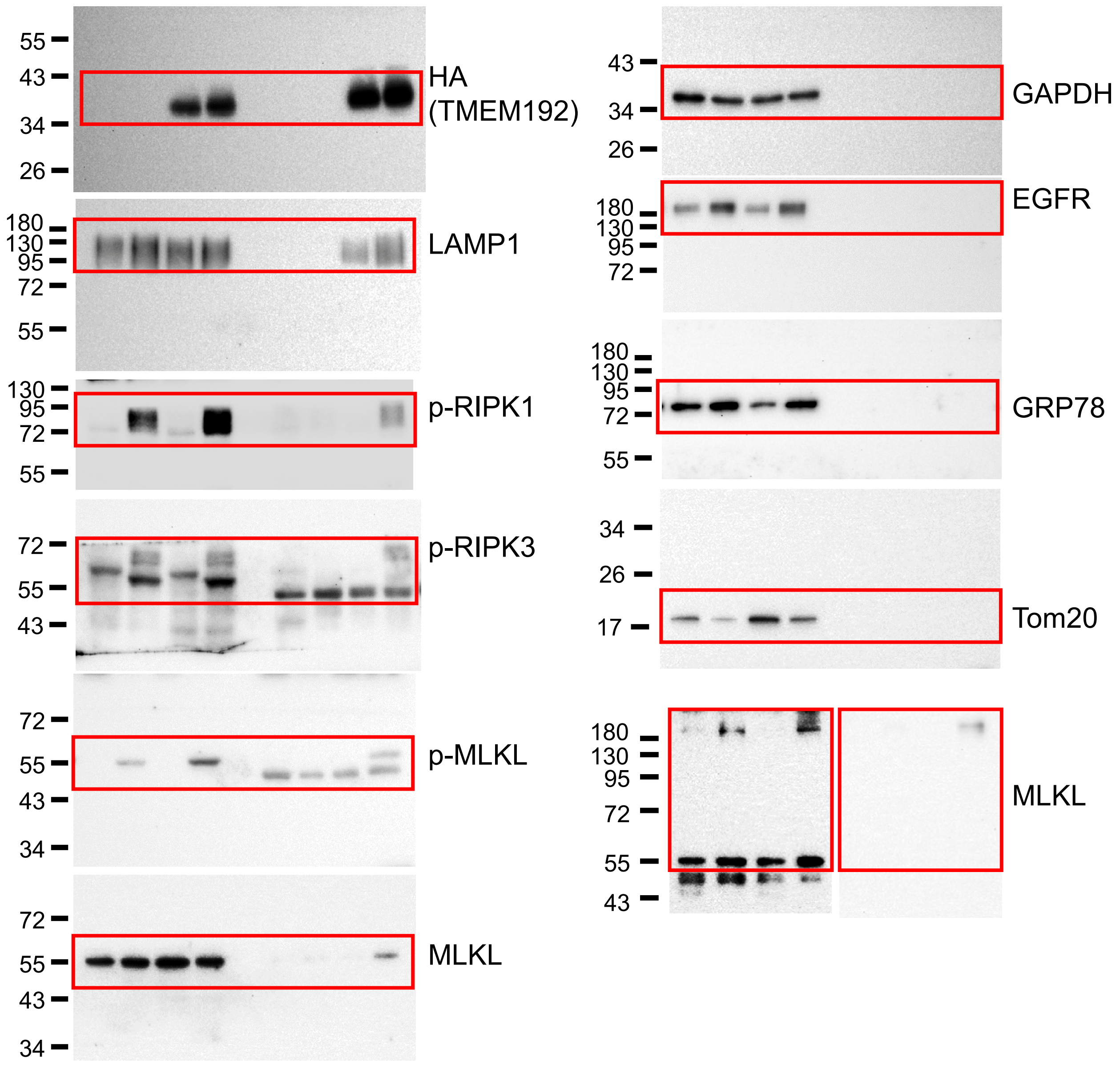

Fig. 2c

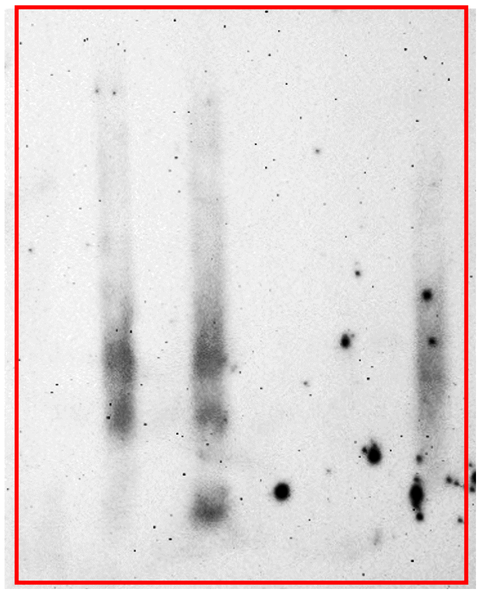

Supplemental Material to Fig. 3 (original blots)

Fig. 3a

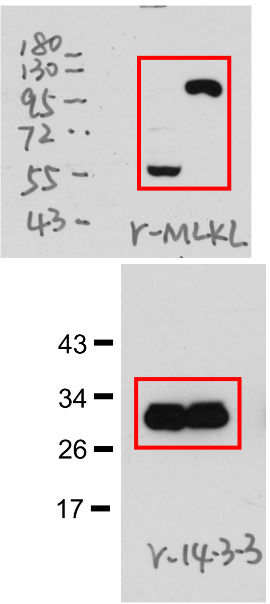

Fig. 3f

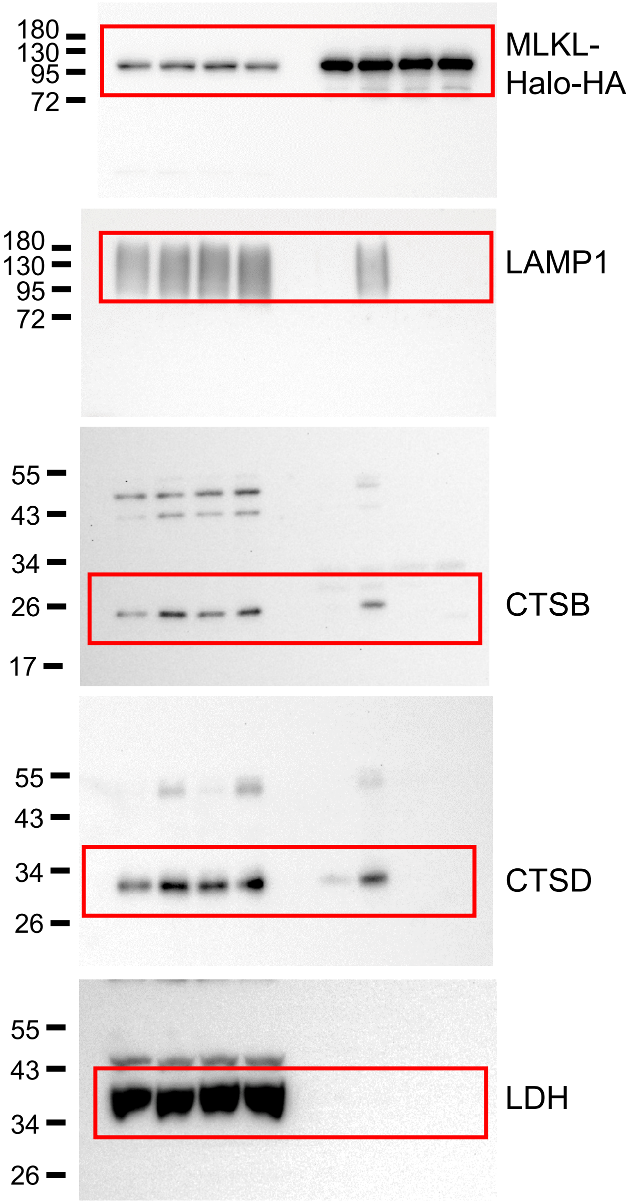

Supplemental Material to Fig. 4 (original blots)

Fig.4b

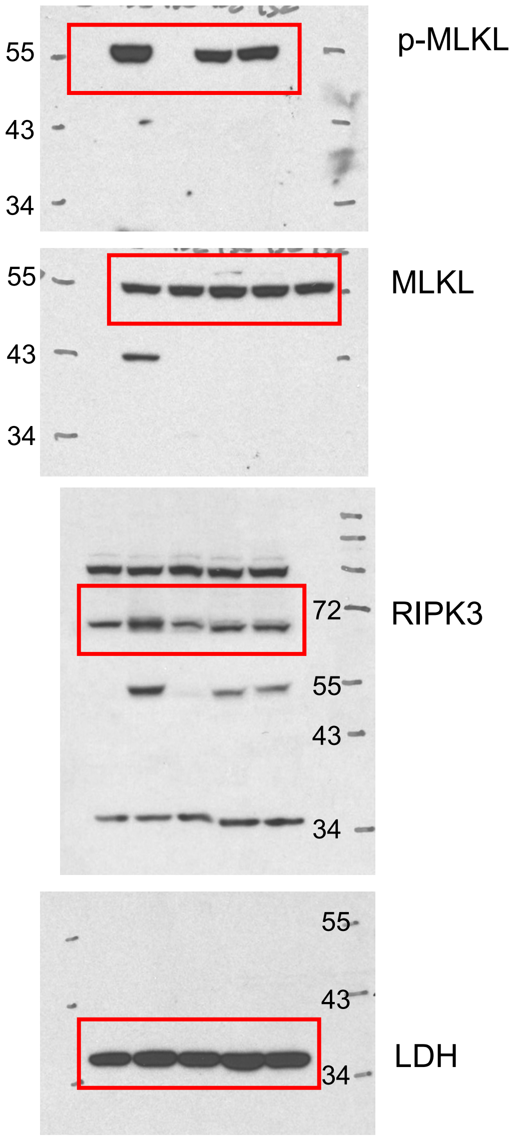

Fig.4c

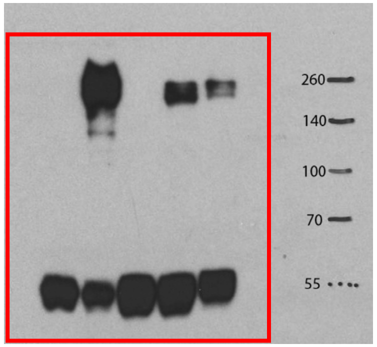

Fig.4d

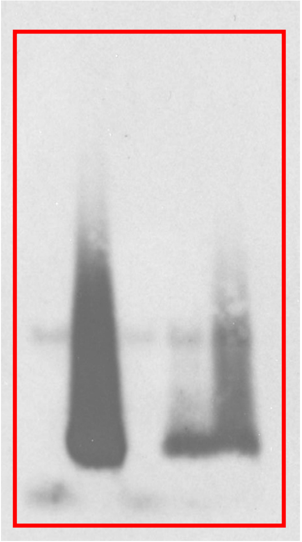

Supplemental Material to Fig. 4 (original blots)

Fig.4e

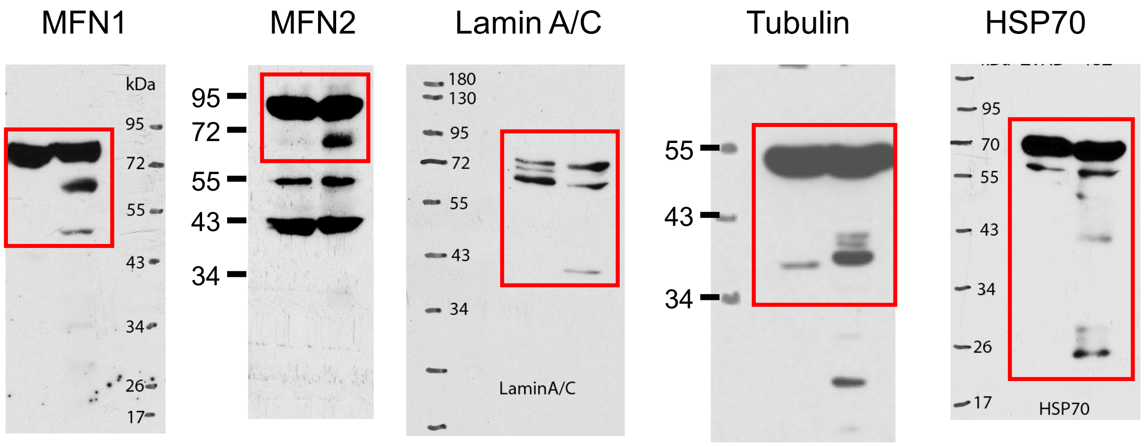

Fig.4f

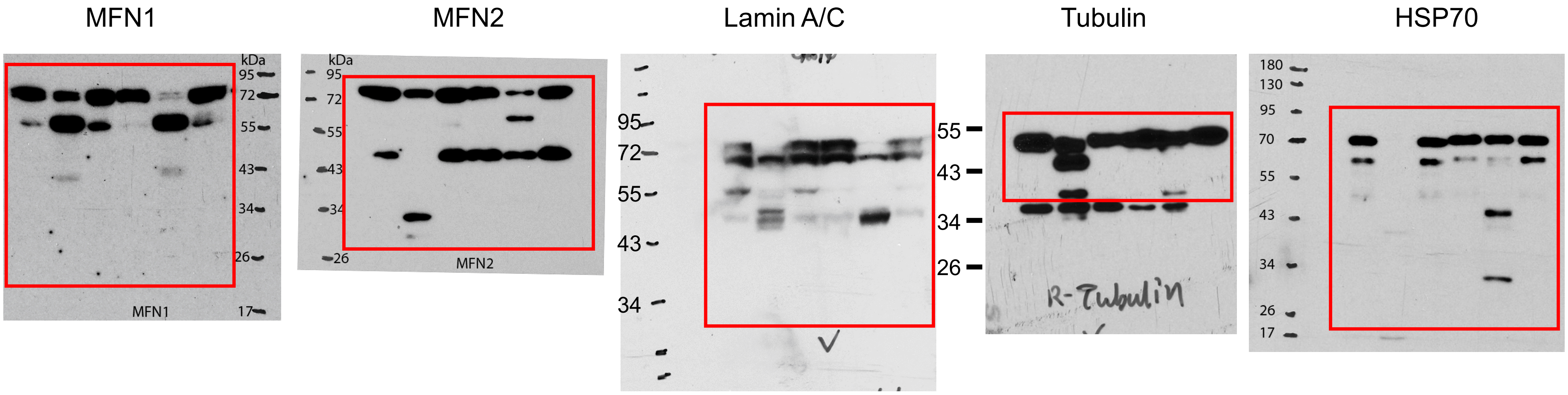

Supplemental Material to Fig. 5 (original blots)

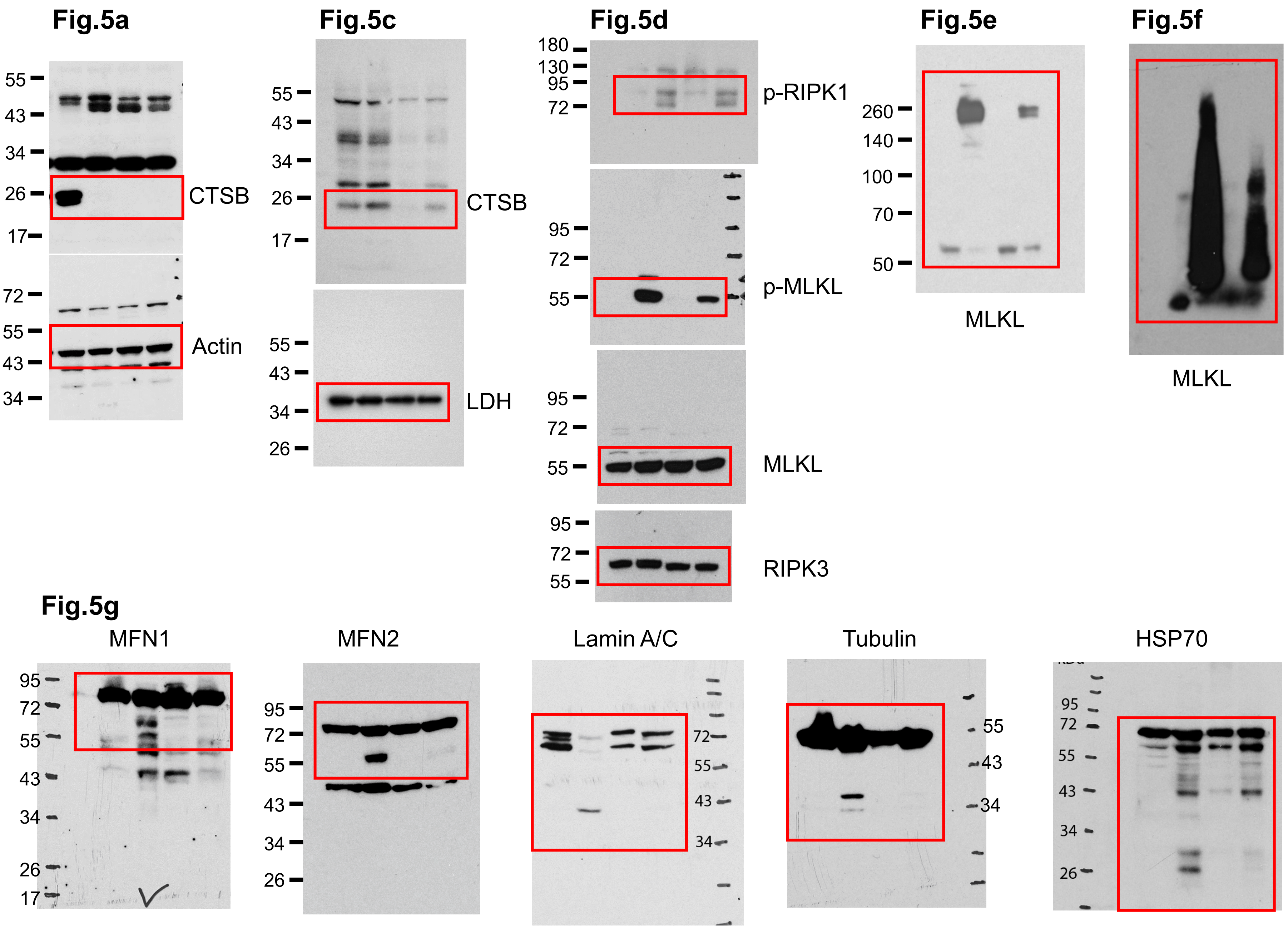

Supplemental Material to Fig. 6 (original blots)

Fig.6c

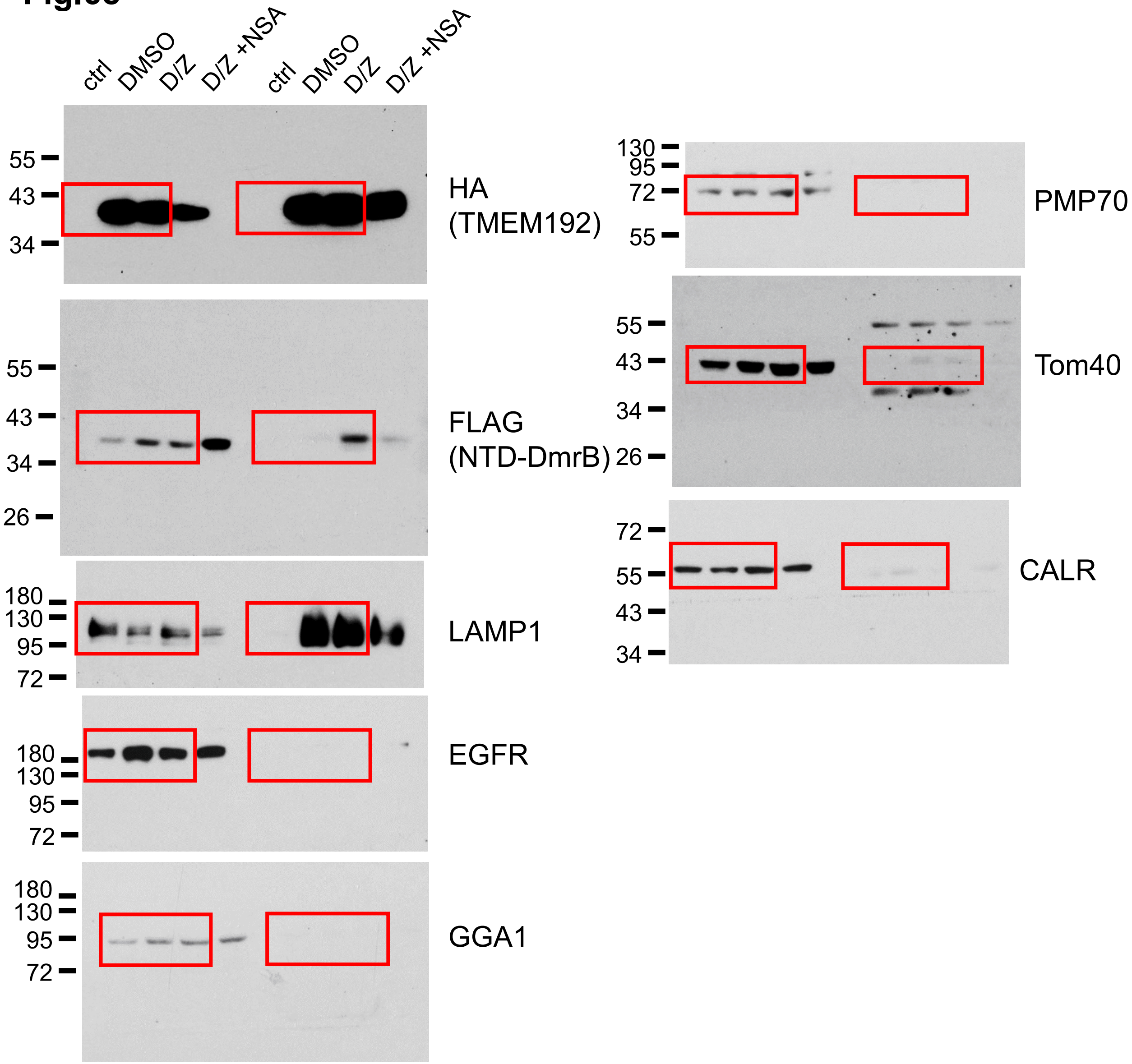

Fig.6d

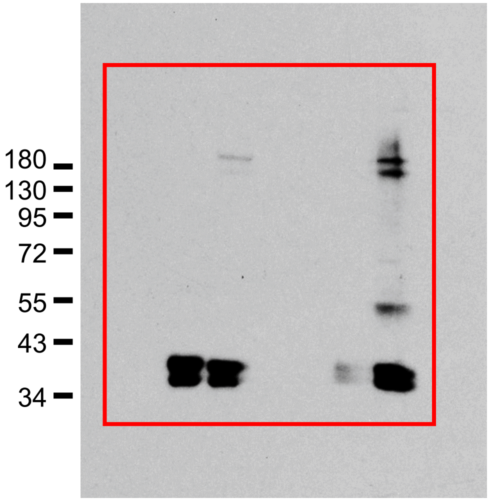

Fig.6e

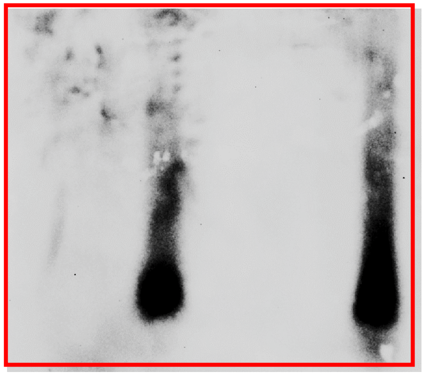

Supplemental Material to Fig. 7 (original blots)

Fig.7d

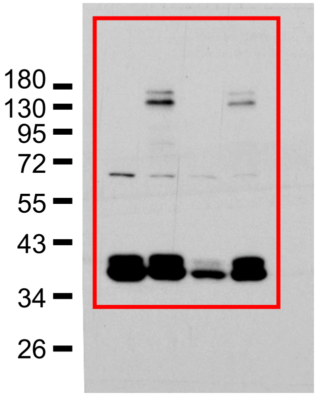

Fig.7e

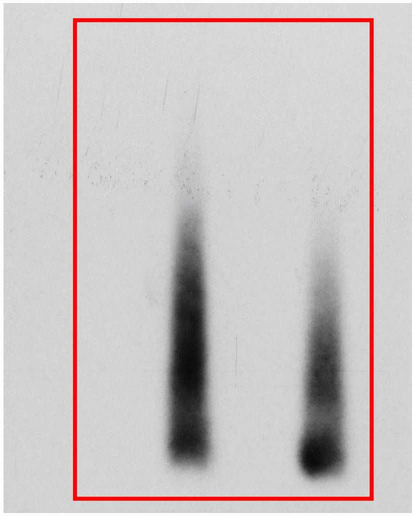

Fig.7f

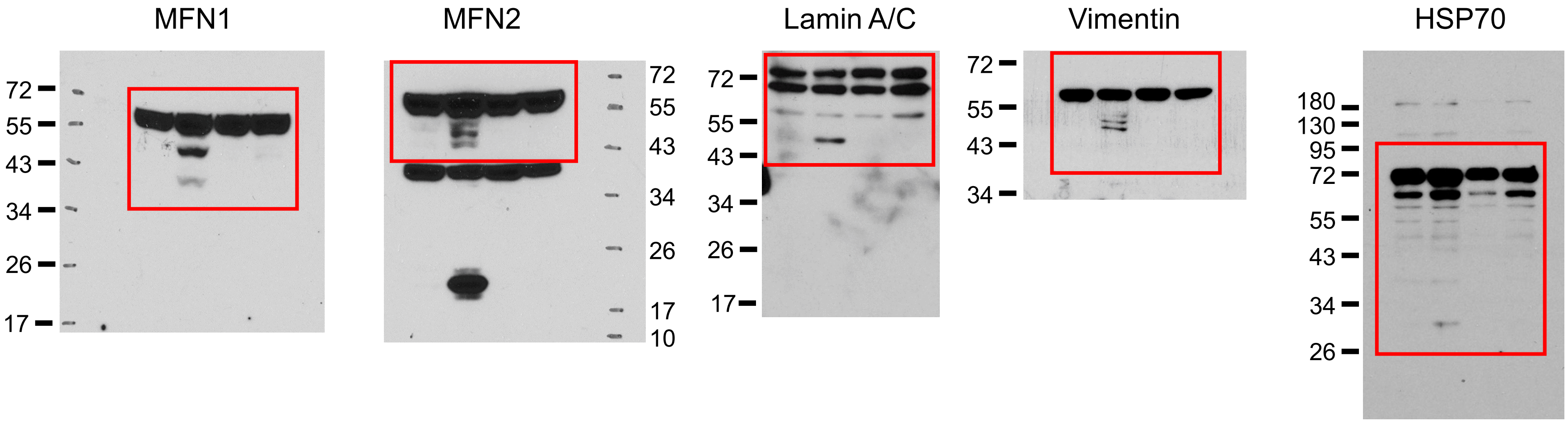

Supplemental Material to Supplemental Fig.1 (original blots)

Fig.S1c

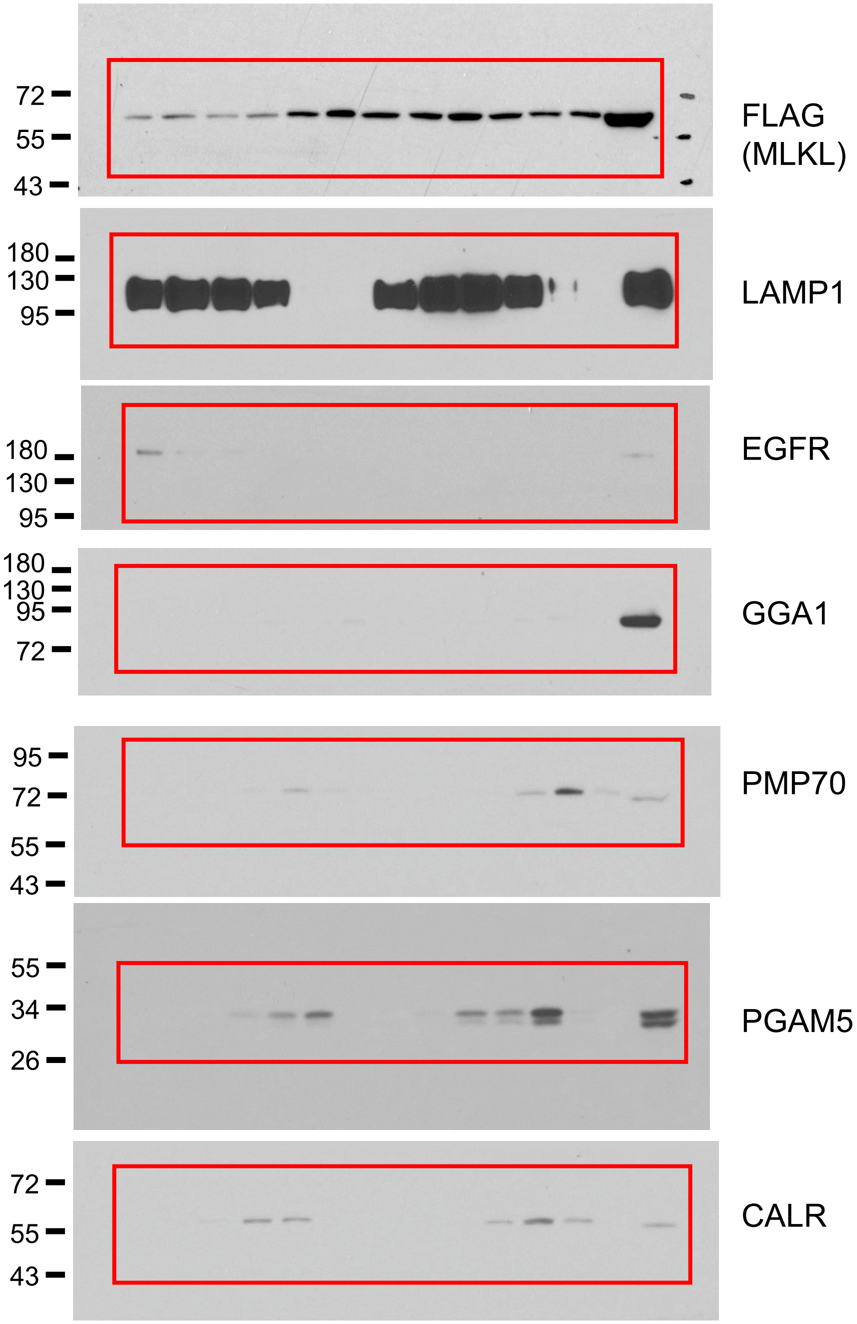

Fig.S1d

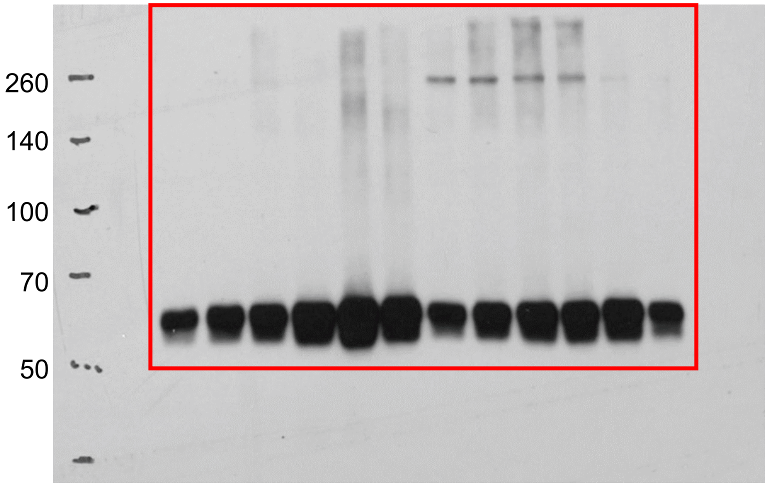

Fig.S1e

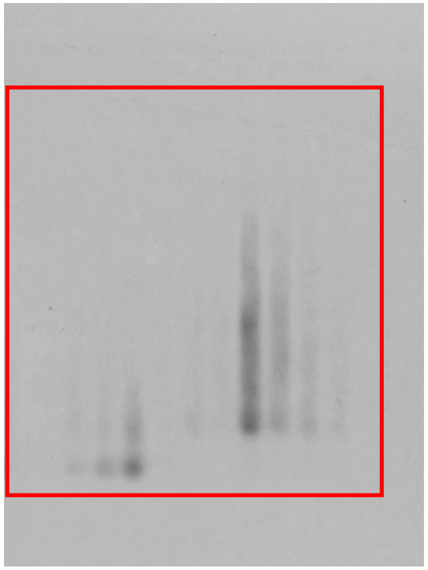

Supplemental Material to Supplemental Fig.3b (original blots)

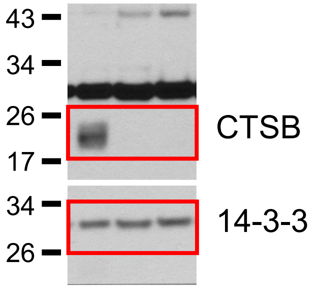

Supplemental Material to Supplemental Fig. 4 (original blots)

Fig.S4b

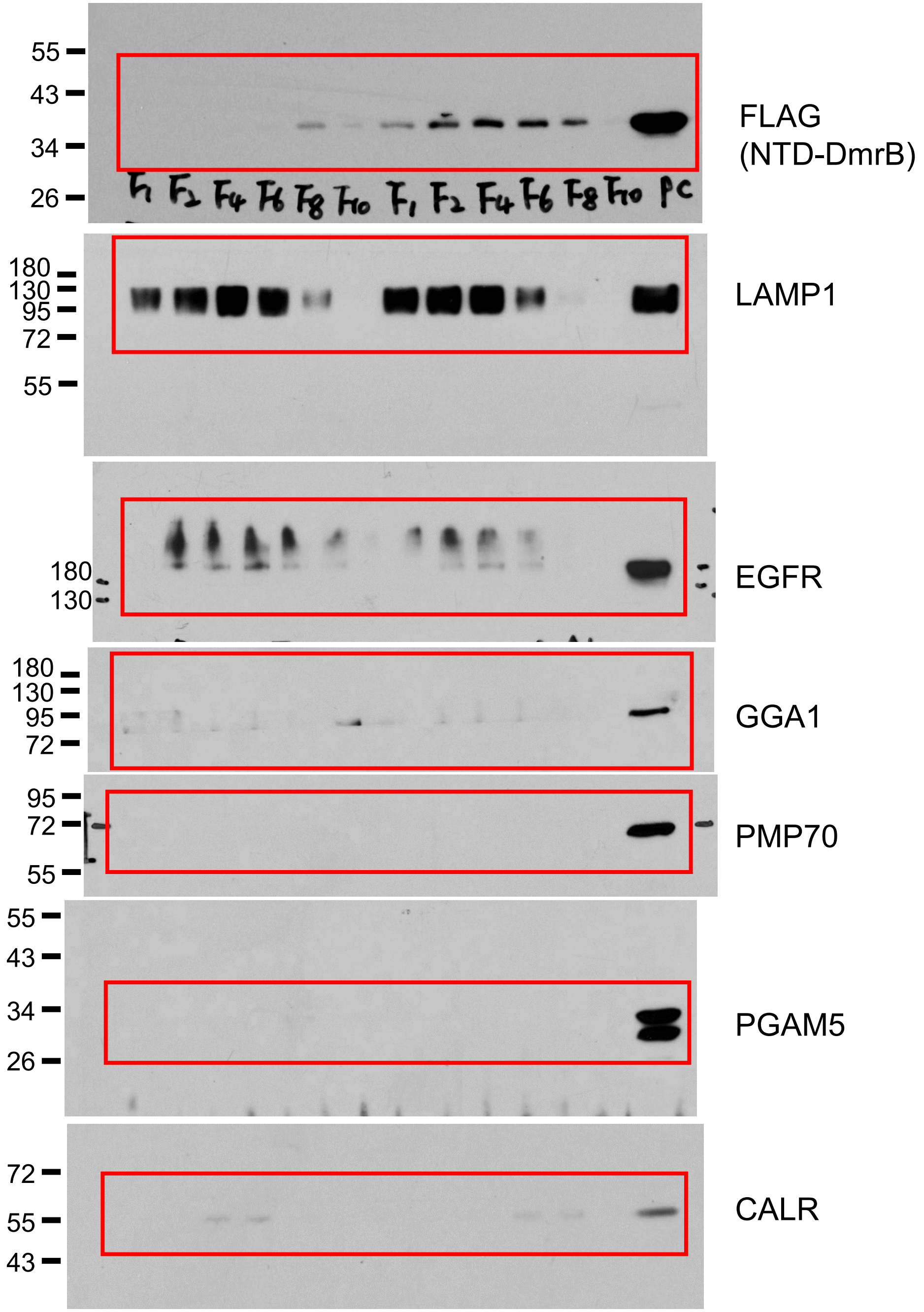

Fig.S4c

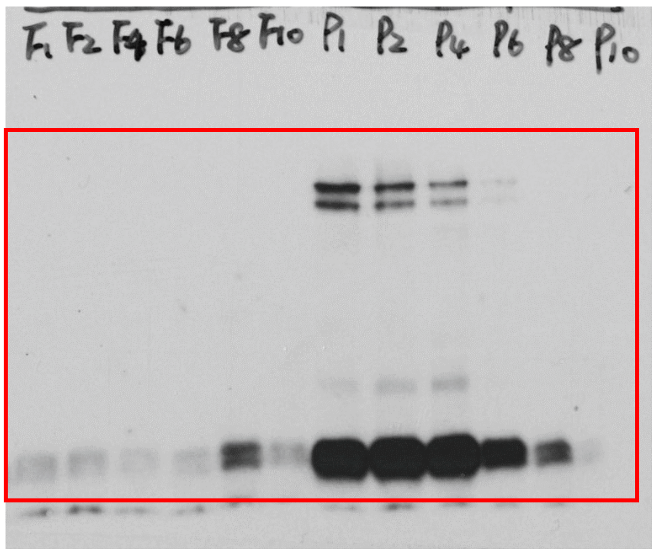

Fig.S4d

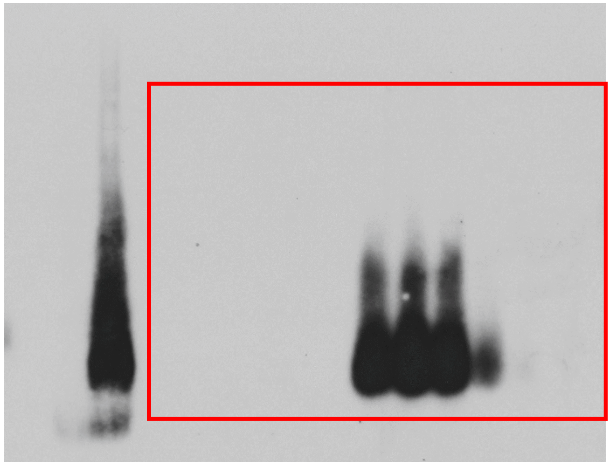

Supplement: Supplementary file 10 — Original experimental data [file 41418_2023_1237_MOESM10_ESM.pdf]
